# Numerical investigation of blood flow effects on temperature distribution in pulmonary tumors during magnetic induction hyperthermia

**DOI:** 10.3389/fbioe.2025.1700261

**Published:** 2025-11-10

**Authors:** Xiao Liu, Mai Lu

**Affiliations:** Key Laboratory of Opto-Electronic Technology and Intelligent Control of Ministry of Education, Lanzhou Jiaotong University, Lanzhou, China

**Keywords:** magnetic induction hyperthermia, blood flow, multi-physics coupling, heat sink effect, temperature distribution

## Abstract

**Introduction:**

Magnetic Induction Hyperthermia (MIH) has emerged as a promising physical approach for tumor treatment and has attracted increasing attention in clinical research. However, most existing studies primarily focus on optimizing magnetic nanoparticle properties and magnetic field parameters, while the heat sink effects induced by blood flow within the treatment region remain insufficiently explored.

**Methods:**

In this study, a three-dimensional lung tumor model incorporating vascular structures and laminar blood flow was established based on multiphysics coupling theory. Using the COMSOL Multiphysics finite element platform, the coupled magnetic, electric, and thermal fields during MIH treatment were simulated to analyze the influence of blood flow on temperature distribution.

**Results:**

Simulation results demonstrated that, without blood flow, the tumor center temperature rapidly increased to 47.7 °C within 300 s, and the peripheral temperature remained above 42 °C, achieving effective hyperthermia. In contrast, when blood flow was introduced, the heat sink effect significantly reduced the therapeutic temperature—the tumor center dropped to 44.5 °C, and the minimum peripheral temperature decreased to 39.4 °C. Both blood flow velocity and vessel diameter were found to strongly influence the heat sink , with lower flow velocity or smaller vessel diameter will mitigate the effect.

**Discussion:**

This study suggests that temperature uniformity during magnetic induction hyperthermia (MIH) can be improved by increasing the number of Helmholtz coil turns, enhancing the excitation current, or optimizing the distribution of magnetic fluid within the tumor region. These findings provide theoretical insights into the role of blood flow in MIH and offer practical guidance for individualized clinical treatment planning.

## 1 Introduction

Malignant tumors represent a major global public health issue. According to the latest data, in 2022, there were 19.976 million new cases and 9.744 million deaths from malignant tumors worldwide. In China, there were 4.825 million new cases, accounting for 24.2% of the global total, and 2.574 million deaths, accounting for 26.4% of global deaths (Chen et al., 2023). Among various cancers, lung cancer has consistently ranked first in both incidence and mortality rates (Bray et al., 2024). With the aging population, environmental pollution, and changing lifestyles, the incidence of malignant tumors continues to rise annually. Lung cancer remains the leading type of solid tumor, with its high rate of incidence, often presenting no obvious symptoms in the early stage, leading to diagnoses at advanced stages. Traditional treatment methods for lung cancer are limited in efficacy, with a relatively low 5-year survival rate (Han et al., 2024).

Currently, standard treatments for lung tumors include surgery, chemotherapy, radiation therapy, and targeted drug therapies. Surgery is the most direct treatment for early localized lesions, but most patients lose the opportunity for surgery by the time of diagnosis. Chemotherapy and radiation can control disease progression, but they have significant side effects, strong resistance, long treatment cycles, and cannot cure the tumor, often causing irreversible damage to normal tissues (Andavar et al., 2025). In recent years, immunotherapy and targeted therapy have made breakthroughs for certain mutated tumor types, but their high costs, large individual variability, and dependence on specific biomarkers limit widespread use. In this context, novel physical, localized, and low-toxicity treatments have become a research focu (Han et al., 2024; Chu and Dupuy, 2014).

Magnetic Induction Hyperthermia (MIH), an emerging non-invasive tumor physical therapy, has shown great promise in experimental and preclinical research. This technique utilizes the sensitivity of tumor cells to high temperatures by introducing magnetic nanoparticles (e.g., Fe_3_O_4_, FeCo) into the tumor region. Under alternating magnetic fields, these nanoparticles generate heat, raising the local temperature above 42 °C, which induces protein denaturation and membrane damage in tumor cells, leading to selective apoptosis while minimizing damage to surrounding normal tissues (Liu et al., 2020; Hildebrandt et al., 2002).

As early as 1957, Gilchrist et al. proposed the concept of selectively heating tumor tissues with magnetic materials in alternating magnetic fields (Gilchrist et al., 1957) Later, the German Jordan team developed MIH equipment in 1993 and successfully validated its therapeutic potential in animal experiments, subsequently developing a heating device with adjustable magnetic field amplitude and frequency (Jordan et al., 1993; Jordan et al., 1999). In China, the team of Tang Jintian made rapid progress in this field, successfully developing an MIH system for clinical research and completing preliminary trial validation (Lmg-Yun et al., 2013). In 2010, the Su team constructed a 3D heat transfer model of tumor tissue, revealing the impact of magnetic particle volume fraction and concentration gradients on the temperature field in the target area (Su et al., 2010). In 2021, the Yu team analyzed how material properties, size, and surface modifications affect the therapeutic effect, providing a theoretical basis for the development of magnetic hyperthermic materials (Yu et al., 2021).

Despite the growing maturity of MIH technology, current research mainly focuses on magnetic material types, injection concentration, heating power, and coil structure optimization. In clinical applications, several challenges remain, one of the key issues being the “heat sink effect,” which weakens the heating effect.

Studies have shown that, under physiological conditions, tumor tissues are often richly vascularized, particularly in highly perfused organs such as the lungs and liver (Ricci-Vitiani et al., 2010). Blood flow can significantly dissipate the heat generated during treatment, forming local heat dissipation zones. This “heat sink effect” not only prevents certain tumor areas from reaching the required therapeutic temperature but also may lead to uneven heating, reduced treatment efficacy, and even treatment failure (Zorbas and Samaras, 2015). Particularly when blood vessels directly pass through the tumor core, rapid heat loss can cause local temperatures to fall far below the target, preventing effective tumor cell apoptosis. Therefore, quantifying and predicting the impact of blood flow on temperature distribution is a critical issue that needs to be addressed for the clinical application of MIH.

Based on this background, this study constructs a 3D lung tumor model with typical vascular structures and performs multi-physics coupling of magnetic, electric, bioheat, and blood flow dynamics models. Using the COMSOL Multiphysics simulation platform, we simulate the temperature distribution under different blood flow velocities and vessel diameters during MIH treatment. Additionally, a Helmholtz coil is introduced as a magnetic field source, allowing for quantitative control over the magnetic field uniformity and strength in the heating region. This study aims to uncover the true impact of blood flow on MIH heating efficacy and propose optimization paths based on numerical simulation to enhance the temperature control accuracy and treatment safety of MIH, providing theoretical support and engineering guidance for the development of future personalized thermal treatment plans.

## 2 Principles and methods

### 2.1 Hemodynamics and mathematical models

Hemodynamics is an important branch of biomechanics that aims to study the flow patterns and mechanical properties of blood within blood vessels. It integrates theories and methods from fluid mechanics, biology, and medicine, with the goal of revealing the physiological and pathological mechanisms of the circulatory system (Qiao and Liu, 2008).

In hemodynamic studies, constructing a unified model that comprehensively describes the blood flow patterns in arterial vessels is crucial. This requires a consideration of the geometric characteristics of the vessels, including their size and shape, while appropriately setting initial and boundary conditions. Moreover, selecting an appropriate constitutive equation for blood is also a key step in model construction, as it must reflect the fluid characteristics and flow state of blood. During the model construction process, it is essential to ensure that the governing equations adhere to the basic physical laws of mass conservation and momentum conservation (Tzirtzilakis, 2005). Because blood is a complex fluid with varying components, when determining the model parameters and equations, it is necessary to balance and select based on the focus and complexity of the specific research problem, to ensure the accuracy and applicability of the model.

#### 2.1.1 Blood fluid properties

In fluid dynamics, fluids can be classified into Newtonian and non-Newtonian fluids. Newtonian fluids are those that obey Newton’s law of viscosity, where the shear stress is linearly related to the shear rate. Common examples of Newtonian fluids include water, alcohol, and glycerin at room temperature. On the other hand, non-Newtonian fluids are those in which the shear stress does not have a linear relationship with the shear rate. The viscosity of non-Newtonian fluids changes with variations in shear stress, exhibiting more complex flow characteristics. Most polymers and solutions composed of polymers are typically considered non-Newtonian fluids (Gertsenshteĭn et al., 2009).

**FIGURE 1:**
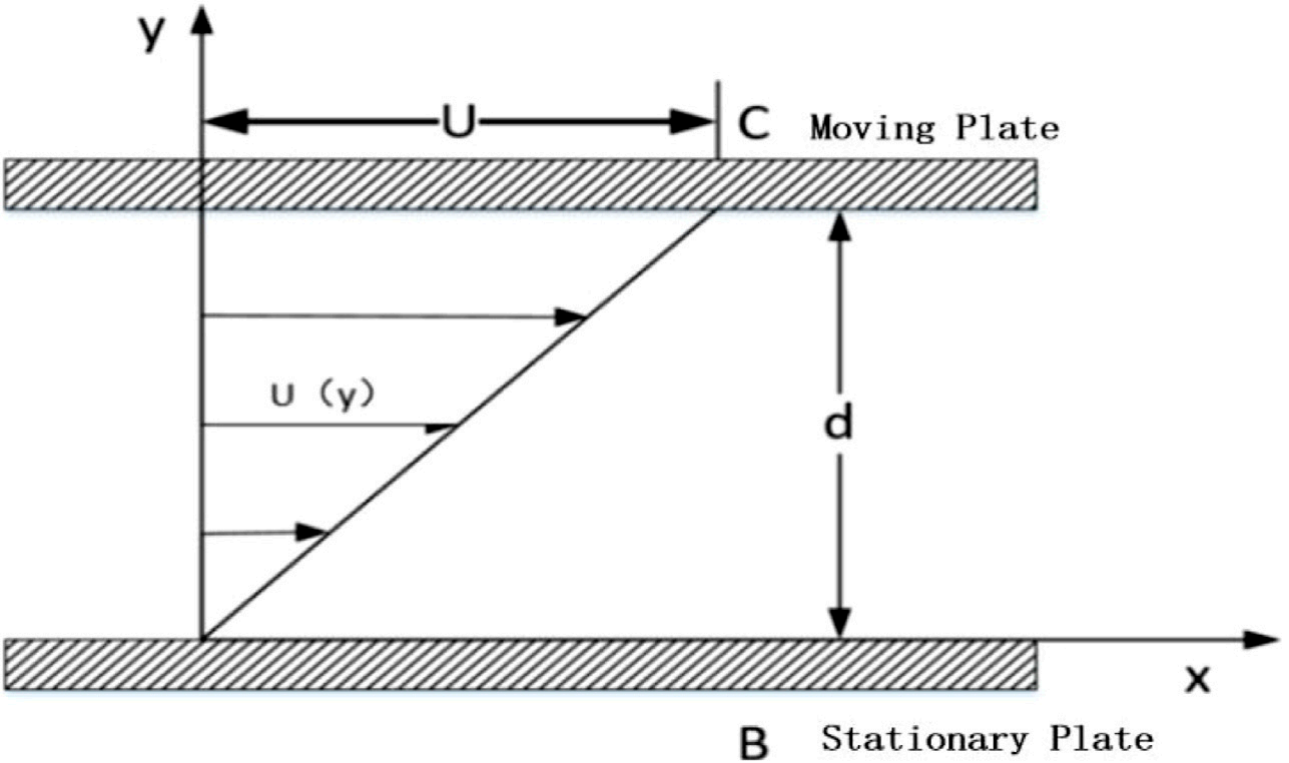
Newton’s parallel plate experiment.

The Newton’s parallel plate experiment suggests that if there are two plates placed close to each other, with fluid filling the space between them as shown in Figure 1, where plate B remains stationary and plate C moves at a constant velocity U under the action of a pulling force F, the fluid between the plates will move in the direction of the moving plate. The fluid velocity decreases as the distance from the moving plate increases. Let the contact area of the moving plate with the fluid domain be A. Then, the shear force F exerted on the fluid by the plates is proportional to the velocity gradient of the moving plate C, and inversely proportional to the distance between the laminar layers. From this, we canderive:

$$\frac{F}{A}=\mu \frac{u}{h}$$ (1)

Where μ is the dynamic viscosity of the fluid, with units of Pas, and h represents the distance between the moving plate C and the laminar flow.

In finite element analysis, it can be expressed as:

$$\tau =\mu \frac{\text{du}}{\text{dy}}$$ (2)

τ represents the shear stress per unit area, with units of N/m^2^. From Equations 1, 2, it can be seen that the viscous force, or the fluid viscosity, is the fundamental cause of flow resistance, and Newtonian fluids satisfy a linear relationship between shear stress and shear strain rate.

In the finite element analysis presented in this study, blood is modeled as aNewtonian fluid for simplicity and ease of analysis.

#### 2.1.2 Blood flow properties

In fluid dynamics analysis, both solid models and 3D simulation models often exhibit certain errors in size and shape compared to the real objects being simulated. In many studies, such as aerodynamics, large objects like aircraft must bescaled down, while for small objects like blood vessels, especially branching vessels, it is necessary to scale up the model. Therefore, the study in reference (Shit and Majee, 2018) points out that the simulation model and the real object must meet the following criteria: geometric similarity, meaning the shape of the model and the boundaries of the fluid must resemble those of the object being simulated; and kinematicsimilarity, which mainly concerns approximating initial and boundary conditions.

Key dynamic parameters in blood flow models often include the Reynolds number (Re). The Reynolds number is an important dimensionless quantity in fluid mechanics, and its physical significance lies in describing the relative magnitude of inertial forces to viscous forces in fluid flow. It is represented as the ratio of inertial forces to viscous forces and is commonly used to determine whether the fluid flow is laminar or turbulent.

The Reynolds number is defined as (Macklin et al., 2009):

$$\text{Re}=\frac{\rho \text{VL}}{\mu }=\left(\rho V^{2}\right)/\left(\mu V/L\right)=\frac{\text{inertial}\;\text{force}}{\text{viscous}\;\text{force}}$$ (3)

Where ρ is the fluid density, in kg/m^3^, V is the average fluid velocity, in m/s, L is the characteristic length (such as the diameter of a pipe or the size of an object),in m, and μ is the dynamic viscosity of the fluid,in Pa.s. When the Reynolds number is small, viscous forces dominate, and the fluid flow remains stable, exhibiting laminar flow. When the Reynolds number is large, inertial forces dominate, causing the fluid flow to become unstable and potentially leading to turbulence. The Reynolds number is a key parameter for determining the flow state of a fluid. Typically, if theReynolds number is less than 2,300, the flow is laminar; if it exceeds 4,000, the flow is turbulent; and values between 2,300 and 4,000 represent transitional flow.

According to references (Zhao et al., 2019; Pirola et al., 2017), the average flow velocity of arterial blood inthe human body ranges from 0.01 to 0.5 m/s, depending on the vessel location and diameter. The dynamic viscosity of blood is 0.005 Pa s, and for this calculation, the vessel diameter is taken as 20 mm, with a blood density of 1,050 kg/m^3^. Substituting these values into Equation 3, the Reynolds number for blood flow is calculated to be 2,100. Therefore, under these conditions, the blood flow exhibits laminar flow characteristics.

#### 2.1.3 Fundamentals of fluid mechanics modeling for blood flow

Blood, as a biological fluid, exhibits complex rheological properties, and its dynamic behavior follows the fundamental principles of continuum mechanics. When constructing a mathematical model for blood flow, it is essential to consider both the conservation of mass and the conservation of momentum, which correspond to the continuity equation and the Navier-Stokes equation system in fluid dynamics, respectively (Tzirtzilakis and Loukopoulos, 2005).

According to the continuum assumption, the mass flow rate through a control volume per unit time in a steady flow field must satisfy the conservation condition. In Cartesian coordinates, the differential form of the continuity equation can be expressed as:

$$\frac{\partial \rho }{\partial t}+\frac{\partial \left(\rho u\right)}{\partial x}+\frac{\partial \left(\rho v\right)}{\partial y}+\frac{\partial \left(\rho w\right)}{\partial z}=0$$ (4)

In the Equation 4, t represents the time variable, and (u,v,w) denote the velocity components in the x, y, and z directions, respectively. ρ is the blood density. For an incompressible Newtonian fluid model, the blood density ρ can be considered a constant. In this case, the equation is simplified as shown in Equation 5:

$$\nabla \cdot V=\frac{\partial \left(u\right)}{\partial x}+\frac{\partial \left(v\right)}{\partial y}+\frac{\partial \left(w\right)}{\partial z}=0$$ (5)

This simplified form has significant practical value in physiological flow analysis, especially in the simulation of blood flow in large vessels of the cardiovascular system.

The Navier-Stokes equations, derived based on Newton’s second law, provide a complete description of the momentum transfer process in viscous fluids. When considering the effects of body forces, The momentum equations in each direction can be expressed as shown in Equations 6–8.

In the X-direction:

$$\frac{\partial \left(\rho u\right)}{\partial t}+\mathrm{div}\left(\rho \text{uV}\right)=\frac{\partial \tau_{\text{xx}}}{\partial x}+\frac{\partial \tau_{\text{yx}}}{\partial y}+\frac{\partial \tau_{\text{zx}}}{\partial z}-\frac{\partial \rho }{\partial x}+f_{x}$$ (6)

In the Y-direction:

$$\frac{\partial \left(\rho v\right)}{\partial t}+\mathrm{div}\left(\rho \text{vV}\right)=\frac{\partial \tau_{\text{xy}}}{\partial x}+\frac{\partial \tau_{\text{yy}}}{\partial y}+\frac{\partial \tau_{\text{zy}}}{\partial z}-\frac{\partial \rho }{\partial y}+f_{y}$$ (7)

In the Z-direction:

$$\frac{\partial \left(\rho w\right)}{\partial t}+\mathrm{div}\left(\rho \text{wV}\right)=\frac{\partial \tau_{\text{xz}}}{\partial x}+\frac{\partial \tau_{\text{yz}}}{\partial y}+\frac{\partial \tau_{\text{zz}}}{\partial z}-\frac{\partial \rho }{\partial z}+f_{z}$$ (8)

Where div represents the divergence, τij denotes the components of the stress acting on the fluid element, in N/m^2^,and $f_{x},f_{y},f_{z}$ represents the components of the body forces in the x, y, and z directions, respectively, acting on the fluid element,in N/m^3^.

### 2.2 Magnetic induction hyperthermia theory and methods

Magnetic Induction Hyperthermia (MIH) is a rapidly developing physical treatment method for tumors, with its core principle being the use of magnetic materials that absorb energy through magnetization in an alternating magnetic field and convert it into heat, thereby raising the temperature of the tumor region to 42–46 °C and selectively inducing apoptosis of cancer cells (Zhang and Lu, 2024). Compared to traditional hyperthermia, MIH offers advantages such as strong targeting, minimal side effects, and the ability to penetrate deep tissues. It has achieved significant progress in the treatment of solid tumors such as glioblastoma and prostate cancer. For nanoscale super paramagnetic particl-es (e.g., Fe_3_O_4_, FeCo), heat generation mainly occurs through relaxation losses; micron-sized ferromagnetic materials (e.g., thermoseeds) rely on hysteresis losses; and millimeter-sized implants (e.g., metal stents) generate heat via eddy current effects (Dennis and Ivkov, 2013).

#### 2.2.1 Relaxation loss

The heating efficiency of superparamag-netic particles is determined by both Néel relaxation and Brownian relaxation. The effective relaxation time τ satisfies the relationship given in Equation 9 (Suriyanto et al., 2017):

$$\frac{1}{\tau }=\frac{1}{\tau_{B}}+\frac{1}{\tau_{N}}$$ (9)

Where τB is the Brownian relaxation time and τN is the Néel relaxation time (all in units of s).

Brownian relaxation is caused by the physical rotational motion of the nanoparticle magnetic particles within the carrier fluid. The time constant τB is influenced by the fluid’s dynamic volume $V_{H}$ (m3) and the viscosity coefficient of the magnetic fluid η (kg/(ms)). The Brownian relaxation time can be expressed as shown in Equation 10:

$$\tau_{B}=\frac{3\eta V_{H}}{k_{b}T}$$ (10)

$$V_{H}={\left(1+\frac{\delta }{R}\right)}^{3}V$$ (11)

In Equation 11, δ is the thickness of the surface modification layer in m,R is the particle radius, $k_{b}$ is the Boltzmann constant in J/K,and V is the particle volume, in m3.

Néel relaxation arises from the phenomenon of magnetic moment rotation lag within magnetic particles, typically manifested as a phase difference between the direction of the magnetic moment and the applied magnetic field. The Néel relaxation time can be expressed as shown in Equation 12:

$$\tau_{N}=\frac{\sqrt{\pi }}{2}\tau_{0}\frac{\exp\left(\Gamma \right)}{\sqrt{\Gamma }},\Gamma =\frac{\text{KV}}{k_{b}T}$$ (12)

In the equation,k (J/m^3^) is the magnetic anisotropy constant,T is the absolute temperat-ure,the unit is Kelvin, K., and $\tau_{0}$ is the relaxation time constant, with a value of 1 × 10^−9^ s.

Based on classical electromagnetic theory, Rosensweig expressed the formula for the heat power density of a unit volume of magnetic fluid in an alternating magnetic field as (Rosensweig, 2002):

$$P_{0}=\pi \mu_{0}\chi_{0}H_{0}^{2}f\frac{2\pi f\tau }{1+{\left(2\pi f\tau \right)}^{2}}$$ (13)

where H0 (A/m) is the magnetic field strength of the alternating magnetic field, f (Hz) is the magnetic field frequency; τ is the effective relaxation time; and μ0 is the permeability of free space,in unit of H/m. The magnetic susceptibility χ0 can be expressedas:

$$\chi_{0}=\chi_{i}\frac{3}{\xi }\left(\cot h\xi -\frac{1}{\xi }\right)$$ (14)

In Equation 14, $\chi_{i}$ is the initial magnetic susceptibi-lity, which can be expressed as:

$$\chi_{i}=\frac{\mu_{0}\varphi M_{d}^{2}V}{3k_{b}T}$$ (15)

ξ is the Langevin parameter, it can be calculated using Equation 16:

$$\xi =\mu_{0}M_{d}V/k_{b}$$ (16)

From Equation 13, it can be seen that the heating power of the magnetic fluid $P_{0}$ is primarily influenced by the magnetic field strength and frequency, as well as the intrinsic parameters of the magnetic nanoparticles. Here, φ representsthe volume fraction of magnetic nanoparticles in the tumor tissue, and $M_{d}$ denotes their magnetization intensity, with units of A/m.

#### 2.2.2 Bioheat transfer

Heat transfer in biological tissues primarily involves three basic forms: conduction, convection, and radiation (Tunç et al., 2006), all of which follow the principle of energy conservation. During magnetic induction hyperthermia (MIH), magnetic nanoparticles absorb energy in an alternating magnetic field and convert it into heat, which then diffuses to the surrounding tissues. If the transient temperature field T (x,y,z,t) is represented in a three-dimensional Cartesian coordinate system, its variation can be described by the following heat transfer governingequation as shown in Equation 17 (Tunç et al., 2006):

$$\rho c\frac{\partial T}{\partial t}=k\frac{\partial^{2}T}{\partial x^{2}}+k\frac{\partial^{2}T}{\partial y^{2}}+k\frac{\partial^{2}T}{\partial z^{2}}+Q$$ (17)

Where ρ, c, and k represent the tissue density (kg·m^-3^), specific heat capacity (J·kg^-1^·K^−1^), and thermal conductivity (W·m^-1^·K^−1^), respectively, and Q is the heat source term per unit volume.

As a classic model in biological fluid heat transfer modeling, the bioheat transfer equation proposed by Pennes has been widely applied in predicting tissue temperature distributions. This model was established based on experimental measurements of the temperature difference between static forearm tissue and blood, and it extends the traditional heat conduction equation by introducing terms for blood perfusion and metabolic heat generation, more accurately reflecting the heat transport mechanism under physiological conditions. When considering the influence of external heating sources, the Pennes model can be extended as shown in Equation 18 (Pennes, 1948):

$$\rho c\frac{\partial T}{\partial t}=\nabla \cdot \left(k\nabla T\right)+\rho_{b}C_{b}\omega_{b}\left(T-T_{b}\right)Q_{m}+\alpha P_{0}$$ (18)

Where ρ and c are the density and specific heat capacity of blood,˙ Wb is the blood perfusion rate (s^-1^), Tb represents the arterial blood temperature, Qm is the metabolic heat generation per unit volume, $\alpha P_{0}$ denotes the external heat source term, and α is the correction factor (taken as 0.55 in this study), used to adjust for the reduced thermal effect caused by viscosity differences of magnetic fluid particles in the cellular microenvironment (Tang et al., 2017).

When the heat generated by the tissue and the heat carried away by the blood reach dynamic equilibrium, the local temperature tends to stabilize and no longer continues to rise. Since the metabolic heat generation is relatively small, it can be neglected in magnetic induction heating (Shitzer and Kleiner, 1980).

The initial conditions define the temperature distribution of the system at t = 0, serving as the starting point for transient analysis. Considering the human body as a thermal system, in this study, the initial temperature of all tissue regions (including the liver, bile duct, and magnetic fluid distribution areas) is uniformly set to 37 °C:

$$T_{1}\left(x,y,z,0\right)=T_{2}\left(x,y,z,0\right)=37℃$$

Boundary conditions are used to describe the thermal flux continuity at the tissue interfaces. Since the tumor is located deep within the body, the influence of the external environment temperature is considered negligible, and the surface of the torso is set as an adiabatic boundary. At the interface between the tumor and normal tissue, the temperature and heat flux are assumed to be continuous. The boundary conditions are given as shown in Equation 19:

$$T_{1}\left(x,y,z,t\right)=T_{2}\left(x,y,z,t\right)$$

$$k_{1}\frac{\partial T_{1}\left(x,y,z,t\right)}{\partial n}=k_{2}\frac{\partial T_{2}\left(x,y,z,t\right)}{\partial n}$$ (19)

In the equation, $T_{1}$ , $T_{2}$ represent the temperatures of the tumor tissue and normal tissue, respectively, $\frac{\partial T}{\partial n}$ denotes the temperature gradient in the normal direction, and $k_{1}$ , $k_{2}$ are the thermal conductivities of the tumor and normal tissue regions, respectively. To simplify the modeling process, this study does not consider the impact of temperature changes on the material thermal properties.

### 2.3 Principle validation

To verify the accuracy of the theoretical framework and numerical approach employed in this study, the work of Wu et al. (Wu et al., 2015) was reproduced. A three-dimensional spherical model with identical dimensions and material properties to those reported in the reference was constructed to represent tumor and normal tissues. Specifically, the tumor was modeled as a solid sphere with a radius of 5 mm, surrounded by a concentric sphere with a radius of 10 mm to represent normal tissue. It was assumed that the magnetic fluid was uniformly distributed within the tumor, and that both temperature and heat flux were continuous across the tumor–tissue interface.

**FIGURE 2:**
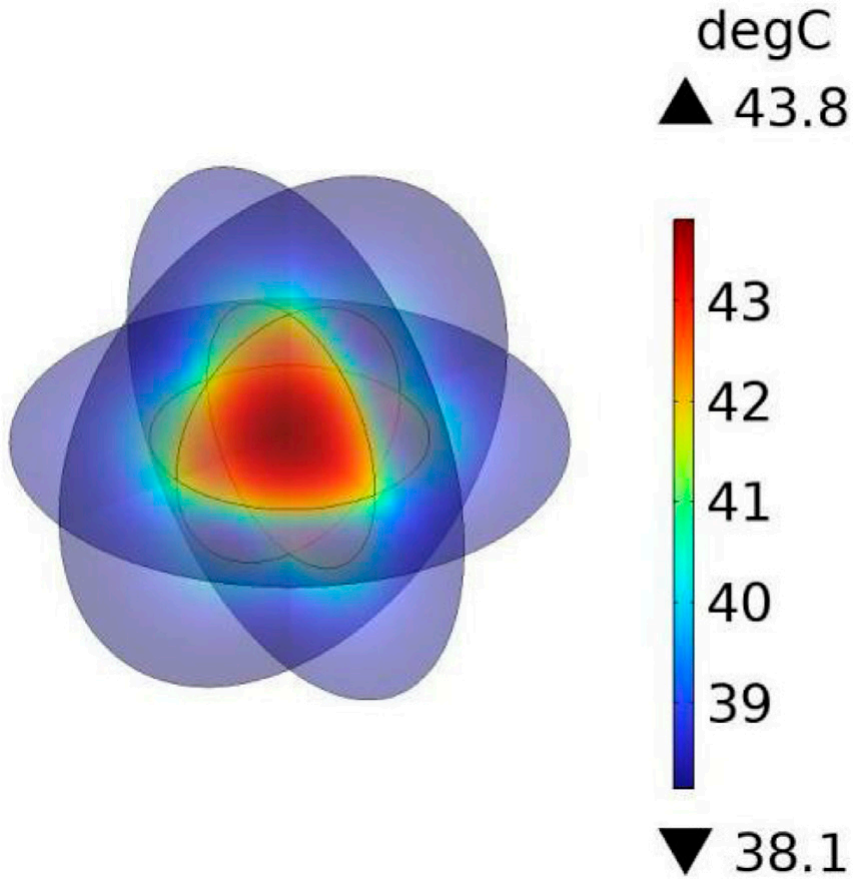
Temperature distribution of the principle validation model.

Under the applied alternating magnetic field (frequency f = 300 kHz, strength H = 5518 A/m), the heating power of the magnetic fluid was calculated as P = 65,720.82 W/m^3^ according to Section 2.1, and this value was used as the heat source in COMSOL to perform transient bio-heat simulations. The results (Figure 2) indicated that the maximum temperature at the tumor center reached 43.75 °C, while the surface of the normal tissue remained at 37 °C. Compared with the reported maximum temperature of 43.47 °C, the deviation was only 0.08 °C, corresponding to an error of approximately 0.6%, thereby demonstrating the reliability of the numerical method adopted in this study.

## 3 Materials and models

### 3.1 Lung cancer model

**FIGURE 3:**
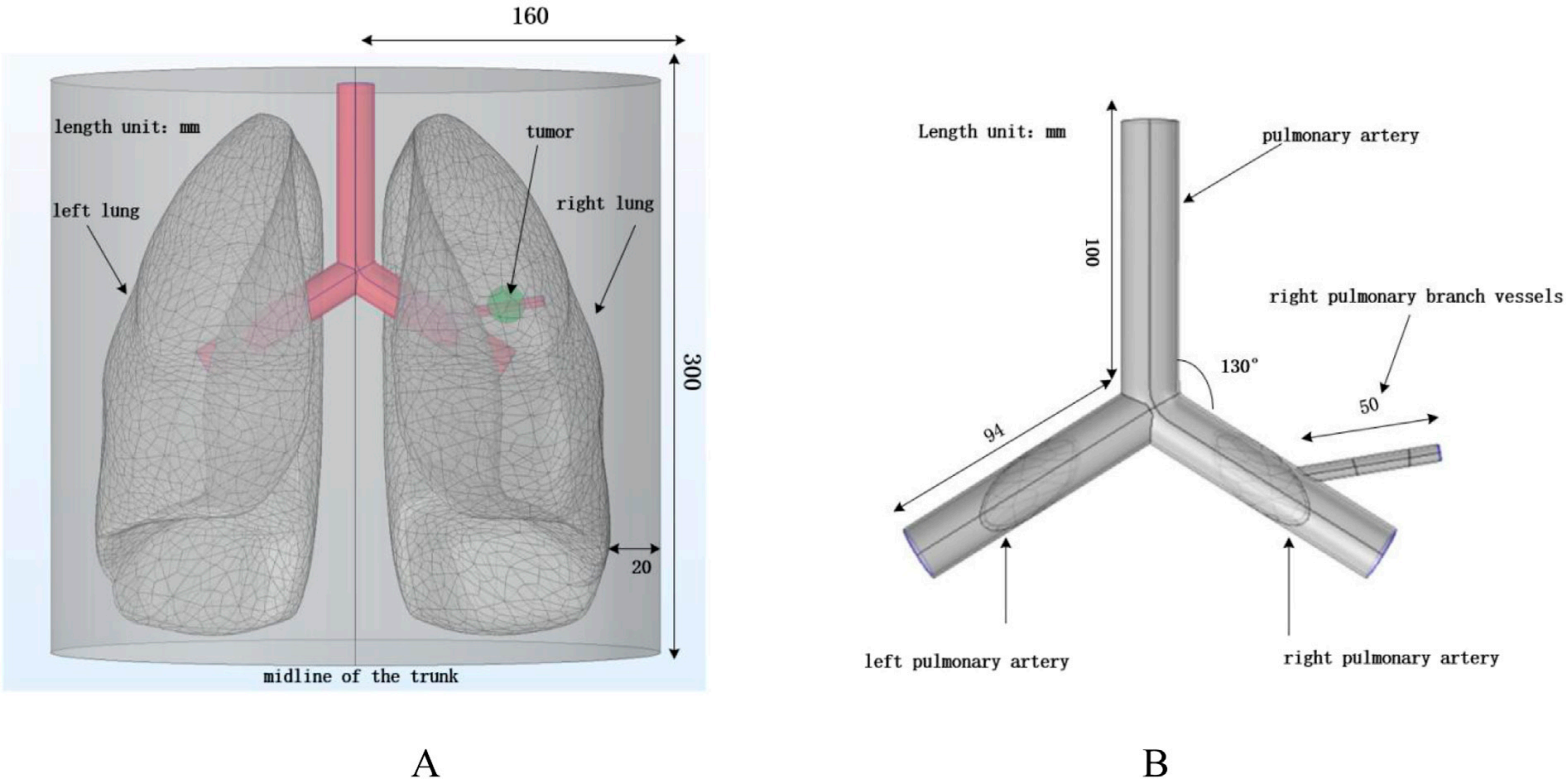
Human lung and vascular model. **(A)** Human thoracic and lung model; **(B)** Vascular structure diagram.

In this study, a simplified three-dimensional lung tissue model of an adult human is constructed, which is further extended to include the chest model and the air domain, as shown in Figure 3. To ensure that the simulation boundaries do not affect the internal magnetic field distribution, the boundaries are set to a magnetic insulation condition. The dimensions of the human lung model are set as follows based on (Wahengbam et al., 2019): length 163 mm, width 260 mm, and height 280 mm.

According to (Townsley, 2012), the human pulmonary artery and the left and right pulmonary arteries are constructed. All three blood vessels have a diameter of 20 mm and a wall thickness of 1 mm. The length of the pulmonary artery is 100 mm, and the lengths of the left and right pulmonary arteries are 94 mm. A solid sphere with a diameter of 20 mm is constructed in the right lung to simulate tumor tissue, while considering the presence of branching blood vessels in the right lung to reflect the impact of blood flow on the heat conduction process. A branch artery with a diameter of 6 mm, wall thickness of 0.4 mm, and length of 50 mm is set in the right pulmonary artery, passing through the tumor domain to simulate the effect of vascular heat sink on the heating process in real scenarios. For convenience, the four arterial blood vessels are placed in the same x-y plane. Laminar flow fluid is set inside the pulmonary arteries to simulate blood flow, and the arteries are treated as rigid cylinders with blood modeled as an incompressible Newtonian fluid (Dong et al., 2021).

### 3.2 Helmholtz coil structure

Magnetic induction hyperthermia commonly uses alternating magnetic field devices, which are mainly divided into magnetic core and coil structures. Compared with magnetic core devices, coil-type devices offer advantages such as simple structure, ease of manufacturing, flexible parameter adjustment, and high space utilization, making them more suitable for use in human treatment environments. However, a disadvantage of coil-type devices is that the magnetic field distribution generated by a single coil is relatively uneven, which makes it difficult to meet the clinical requirement for uniform field strength in hyperthermia (Stauffer et al., 2002). Therefore, in this study, a Helmholtz coil is used as the alternating magnetic field generation device to improve the magnetic field uniformity and enhance the therapeutic effect.

**FIGURE 4:**
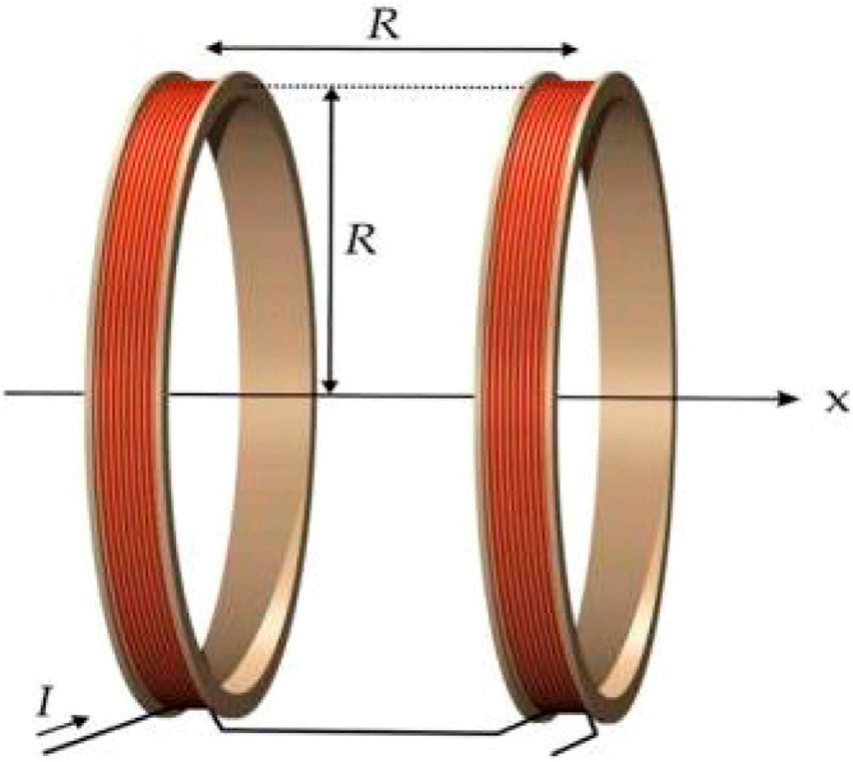
Helmholtz coil structure diagram.

A Helmholtz coil consists of two circular coils with the same radius, placed symmetrically on either side of the central point O, with a spacing equal to the radius R, as shown in Figure 4.

The two coils are connected in parallel with currents flowing in the same direction, creating an approximately uniform magnetic field near the central axis. The uniformity of the magnetic field distribution in a Helmholtz coil is widely applied in geomagnetic compensation, bioelectromagnetic experiments, and thermal therapy devices. According to the Biot-Savart law, the magnetic flux density Bx at any position x along the axis of a single circular coil can be determined using Equation 20:

$$B_{x}=\frac{\mu_{0}\text{NI}R^{2}}{2{\left[R^{2}+x^{2}\right]}^{\frac{3}{2}}}$$ (20)

Where μ0 = 4π × 10–7 H/m is the magnetic permeability of free space, N is the number of turns of the coil, I is the peak current, R is the radius of the coil, and x is the position coordinate along the axis. When considering the superposition of the two coils, according to the principle of magnetic field superposition (Restrepo et al., 2017), the total magnetic induction intensity can be determined using Equation 21:

$$B=B_{L}+B_{R}=\frac{\mu_{0}\text{NI}R^{2}}{2{\left[R^{2}+{\left(\frac{R}{2}+x\right)}^{2}\right]}^{\frac{3}{2}}}+\frac{\mu_{0}\text{NI}R^{2}}{2{\left[R^{2}+{\left(\frac{R}{2}-x\right)}^{2}\right]}^{\frac{3}{2}}}$$ (21)

### 3.3 Heating system

The heating power of the magnetic fluid is influenced by the strength and frequency of the applied magnetic field. The frequencies typically used for hyperthermia range from 10 kHz to 500 kHz. Low frequencies (below 50 kHz) may stimulate the neuromuscular system, while high frequencies may induce strong eddy currents, potentially causing inadvertent damage to healthy tissues (Bohnert et al., 2009). Considering the penetration depth, safety, and therapeutic effects, a frequency of 100 kHz for the alternating magnetic field was selected. The coil size is based on the “Chinese Adult Body Size” (Standards Administration of the People’s Republic of China, 1988), where 99% of men have a shoulder width less than 486 mm, and women have a shoulder width less than 458 mm. To ensure the human body is positioned at the center of the magnetic field and to accommodate the space requirements for the device housing and cooling structure, the coil diameter is set to 600 mm.

**FIGURE 5:**
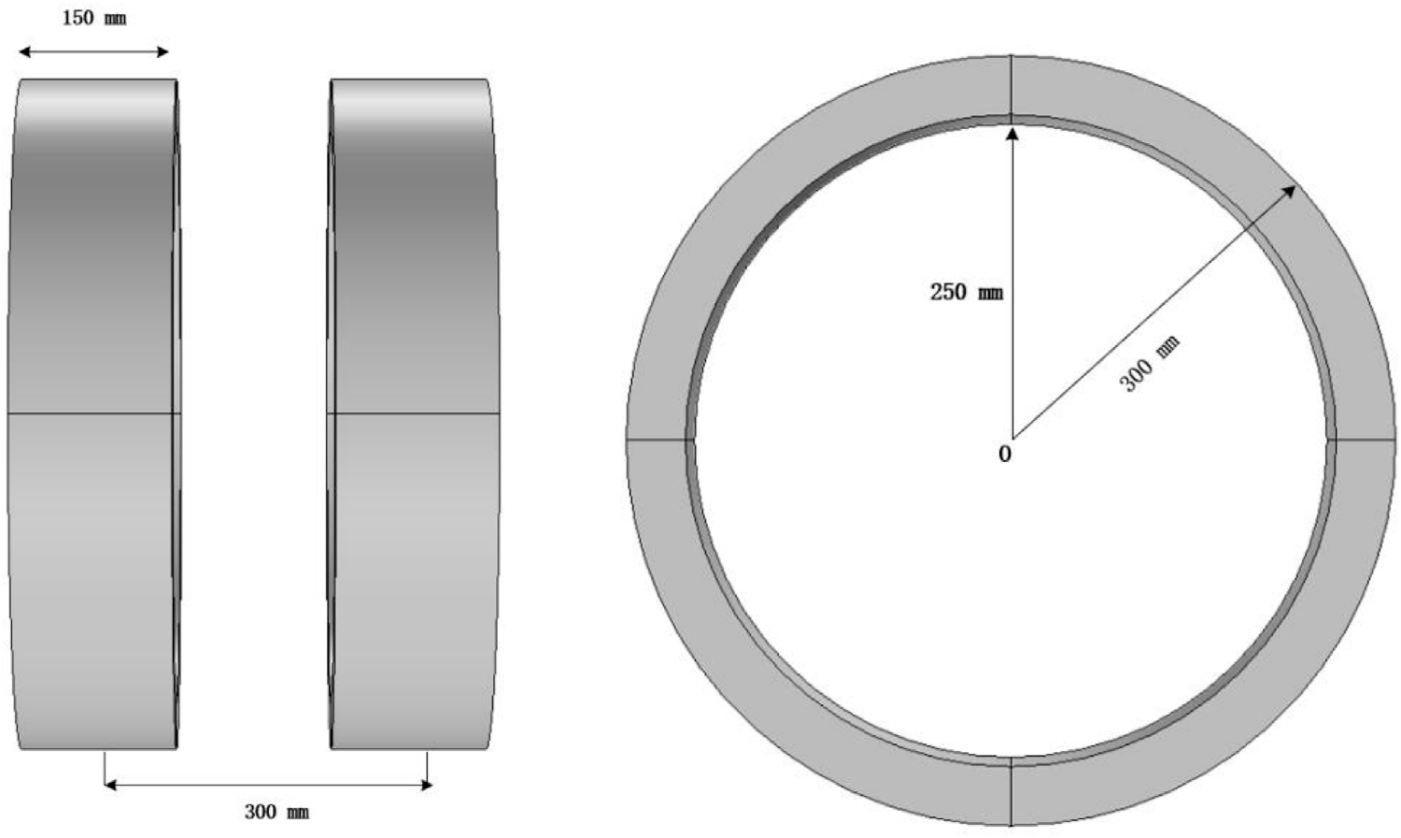
Helmholtz coil model.

The three-dimensional coil model constructed in this study is shown in Figure 5. Both coils have a radius of 300 mm, a length of 150 mm, and a thickness of 50 mm, with a center-to-center distance of 300 mm. The excitation current for the coils is set to I = 10 A, with a coil turn number N = 380 and a frequency of 100 kHz. Copper is chosen as the coil material, with a conductivity of σ = 5.998 × 107 S/m, a relative permeability of 1, and a wire cross-sectional area of 2 mm^2^. The fixed bottom structure and power supply interface are omitted in the model.

**FIGURE 6:**
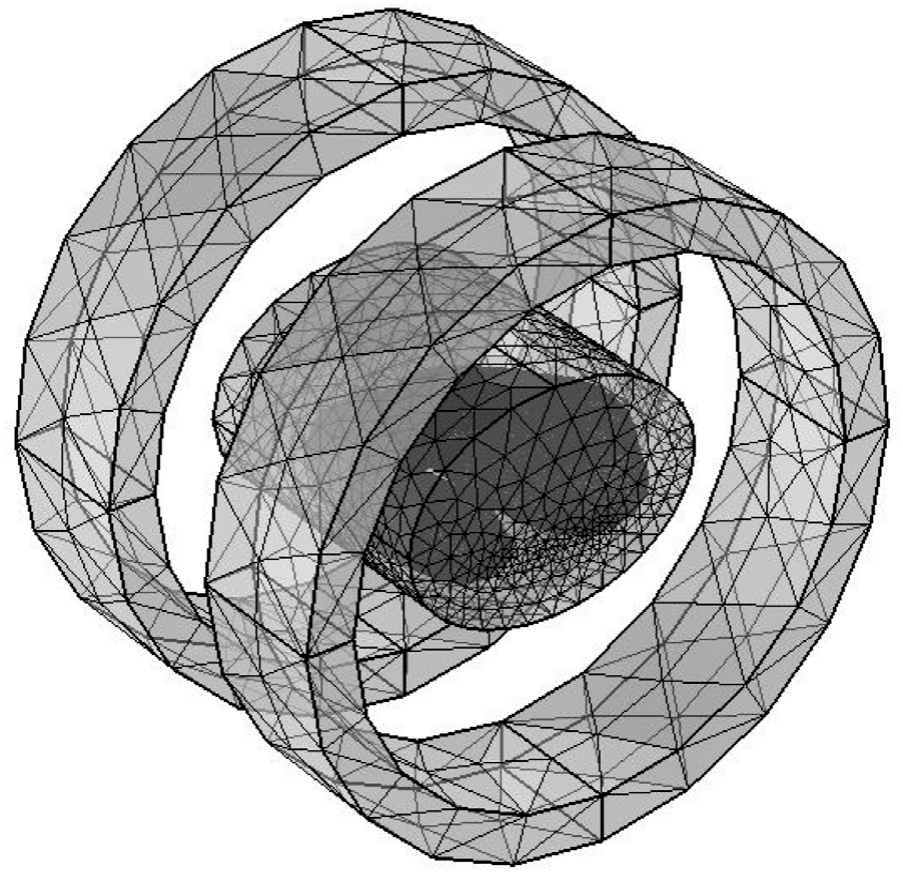
The mesh of coil and human chest model.

**FIGURE 7:**
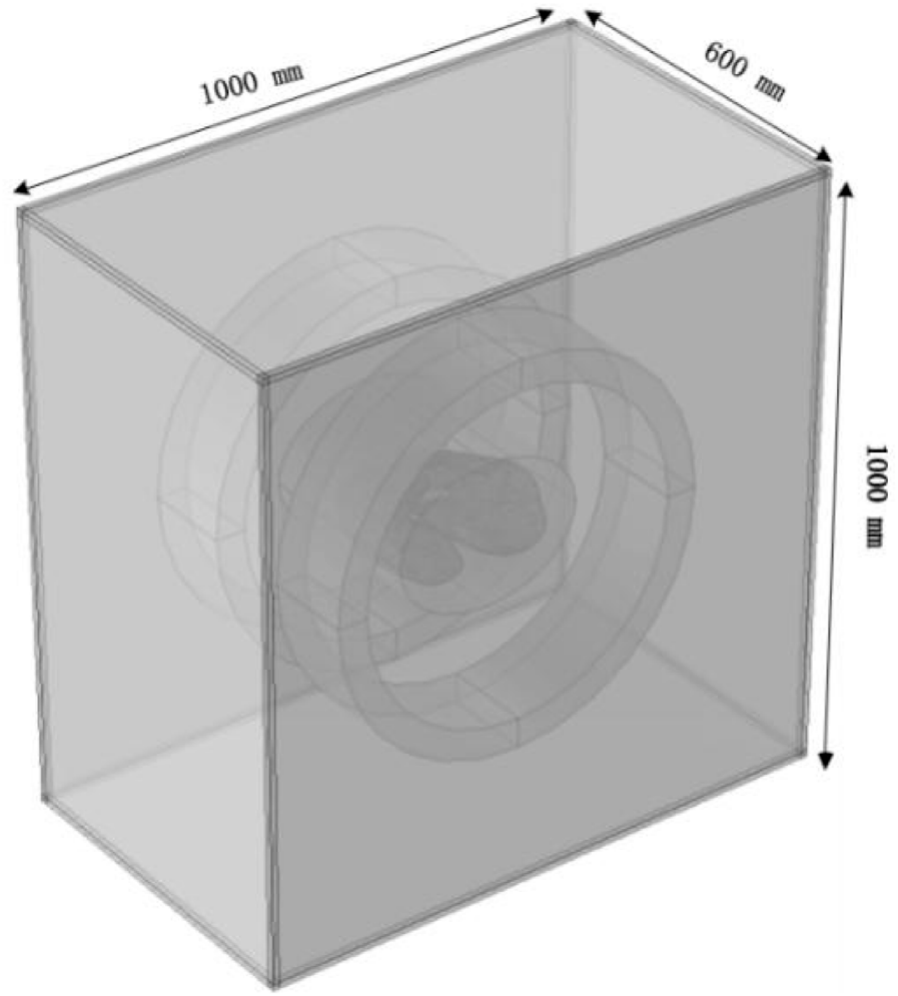
Heating system with added air domain.

The solution domain uses a free tetrahedral mesh with a total of 576,661 elements, where the blood vessel and tumor regions are meshed with finer grids, as shown in Figure 6, to improve local calculation accuracy. The overall perspective model of the heating system after adding the air domain is shown in Figure 7.

### 3.4 Biological tissue parameters

In the magnetic induction hyperthermia process, the distribution of the electromagnetic field in biological tissues is closely related to the dielectric properties of the tissues. Since the dielectric properties of various human tissues exhibit significant differences at different frequencies, to ensure the accuracy of the simulation results, this study uses the classical 4th-order Cole-Cole model (Gabriel et al., 1996) to calculate the dielectric parameters of the tissues. The model is designed for an operating frequency of 100 kHz, which is commonly used in clinical magnetic induction hyperthermia and is suitable for efficient heating of magnetic nanoparticles.

Based on the measured data of different tissues at this frequency (Baumgartner and Hasgall, 2024), the conductivity (σ) and relative permittivity (ϵr) of major tissues, such as human skin, lung tissue, arterial vessel walls, lung tumor tissue, blood, muscle, and bone, were calculated. These parameters were then incorporated into the model for solving the electromagnetic field. Considering the complexity of tissue structures and the necessity of the homogeneity assumption, the average dielectric parameters of skin, muscle, blood, and bone were used as substitutes for the “torso” tissue’s electromagnetic parameters. The dielectric parameters of lung tissue were based on measured data from the human lung in an inflated state, while the parameters for lung tumor tissue were estimated from high-metabolic tumors (such as lung adenocarcinoma). The blood parameters were based on standard laboratory data for arterial whole blood. The resulting dielectric parameters are listed in Table 1.

**TABLE 1:** Dielectric constants and conductivities of various human biological tissues at 100 kHz frequency.

Tissue	Conductivity (S/m)	Relative permittivity (ϵr)
Torso	0.4897	5,933
Lung	0.1070	2,580
Blood	0.7030	5,120
Vessel Wall	0.3190	930

To improve the heating efficiency and temperature distribution uniformity in magnetic induction hyperthermia, this study employs the direct injection method to inject magnetic fluid into the lung tumor tissue, ensuring a more uniform distribution of magnetic nanoparticles within the tumor region. The introduction of magnetic particles will alter the thermophysical properties of the tumor tissue, so the density, specific heat capacity, and thermal conductivity of the mixed tissue need to be corrected.

The equivalent thermophysical parameters of the tumor tissue mixed with magnetic fluid are calculated using a volume fraction-weighted average, and the expression is as follows:

$$\begin{array}{cc} \rho_{2} & =\left(1-\varphi \right)\rho_{1}+\varphi \rho_{3}\\ c_{2} & =\left(1-\varphi \right)c_{1}+\varphi c_{3}\\ \frac{1}{k_{2}} & =\frac{\left(1-\varphi \right)}{k_{1}}+\frac{\varphi }{k_{3}} \end{array}$$

Where ρ1,c1,k1 are the original density, specific heat capacity, and thermal conductivity of tumor tissue; ρ3,c3,k3 are the density, specific heat capacity, and thermal conductivity of the magnetic fluid; ρ2,c2,k2 are the equivalent thermophysical parameters of the magnetic fluid-tumor tissue mixture; and φ is the volume fraction of the magnetic fluid in the tumor, which is set to 0.003 in this study based on clinical standard dosages (Wu et al., 2015). The thermophysical parameters of the biological tissue are shown in Table 2.

**TABLE 2:** Thermophysical parameters of biological tissues.

Tissue	Density ρ (kg·m−3)	Specific heat capacity (J·kg−1)	Blood perfusion rate (1 s−1)
Torso	1,040	3,600	0.0064
Tumor	1,060	3,650	0.0139
Lung	394	3,886	0.00667
Blood	1,050	3,617	—
Vessel	1,102	3,306	—

## 4 Results and analysis

### 4.1 Spatial magnetic field distribution

**FIGURE 8:**
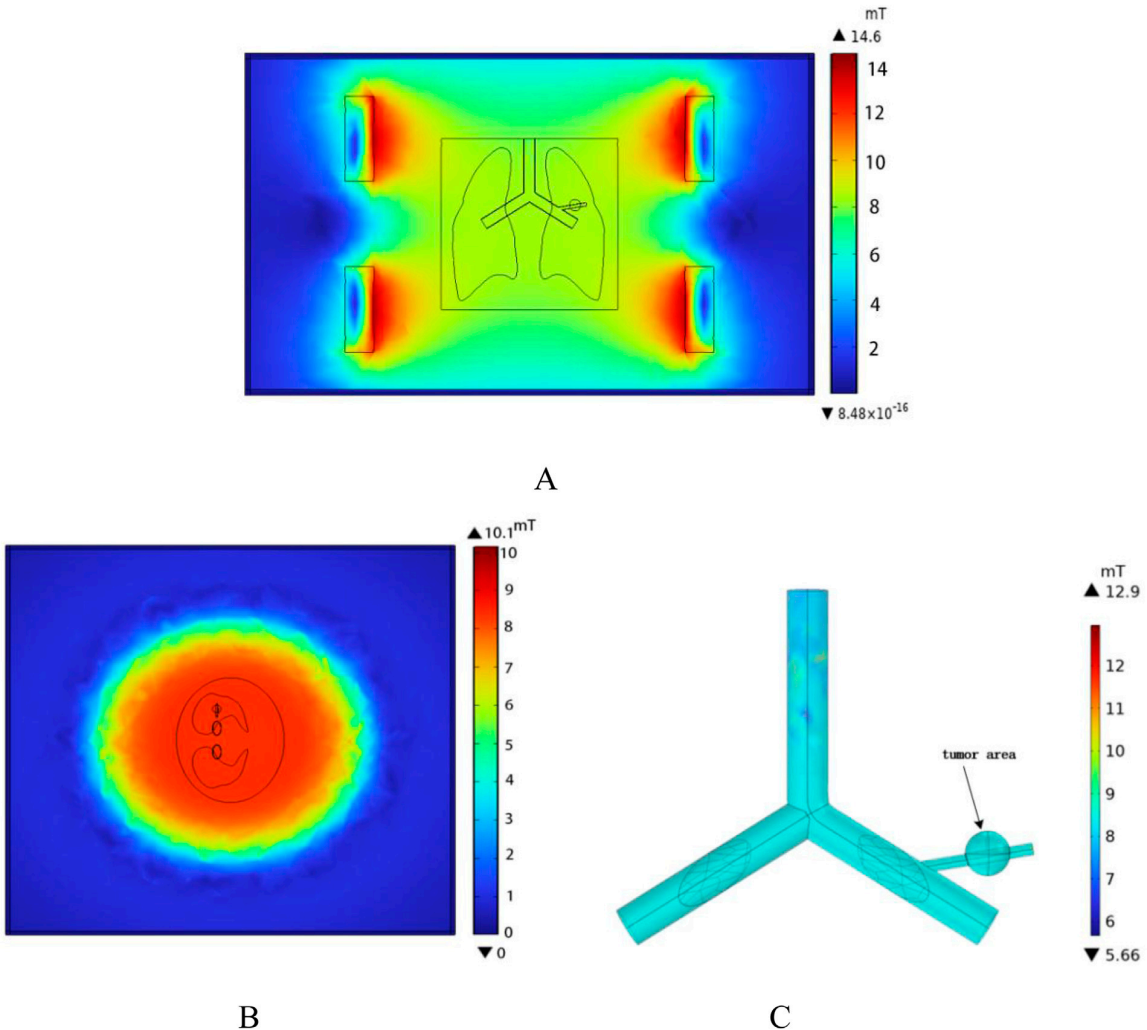
Spatial magnetic field distribution **(A)** x-y plane magnetic field distribution **(B)** z-x plane magnetic field distribution **(C)** Magnetic field distribution in the vessel wall and tumor region.

To ensure that the spatial electromagnetic field generated by the coil meets the requirements for hyperthermia, the spatial magnetic field distribution of the human lung model was computed using the COMSOL Multiphysics finite element calculation software under the conditions of the Helmholtz coil and the previously established parameters. In the case where the Helmholtz coil is used as the magnetic field generation device, with 280 turns of coil, a frequency of 100 kHz, and a coil current of I = 10A, the magnetic induction strength distribution in the x-y plane was calculated, as shown in Figure 8A. According to the results in Figure 8, the magnetic induction strength measured at the edge of the torso is 8.29 mT, and at the center of the chest cavity, it is 8.38 mT. The amplitude change in magnetic induction strength between these two points is 1.07%. This shows that the spatial magnetic field distribution around the torso and lungs is relatively uniform, which meets the requirements for uniformity in magnetic induction hyperthermia. Additionally, the magnetic field strength rapidly decreases outside the coil, which significantly reduces the potential electromagnetic exposure risk for other parts of the patient’s body and medical staff using the device. Furthermore, selecting the z-x cross-section where the lung tumor is located, the spatial electromagnetic field distribution in this section is shown in Figure 8B.

**FIGURE 9:**
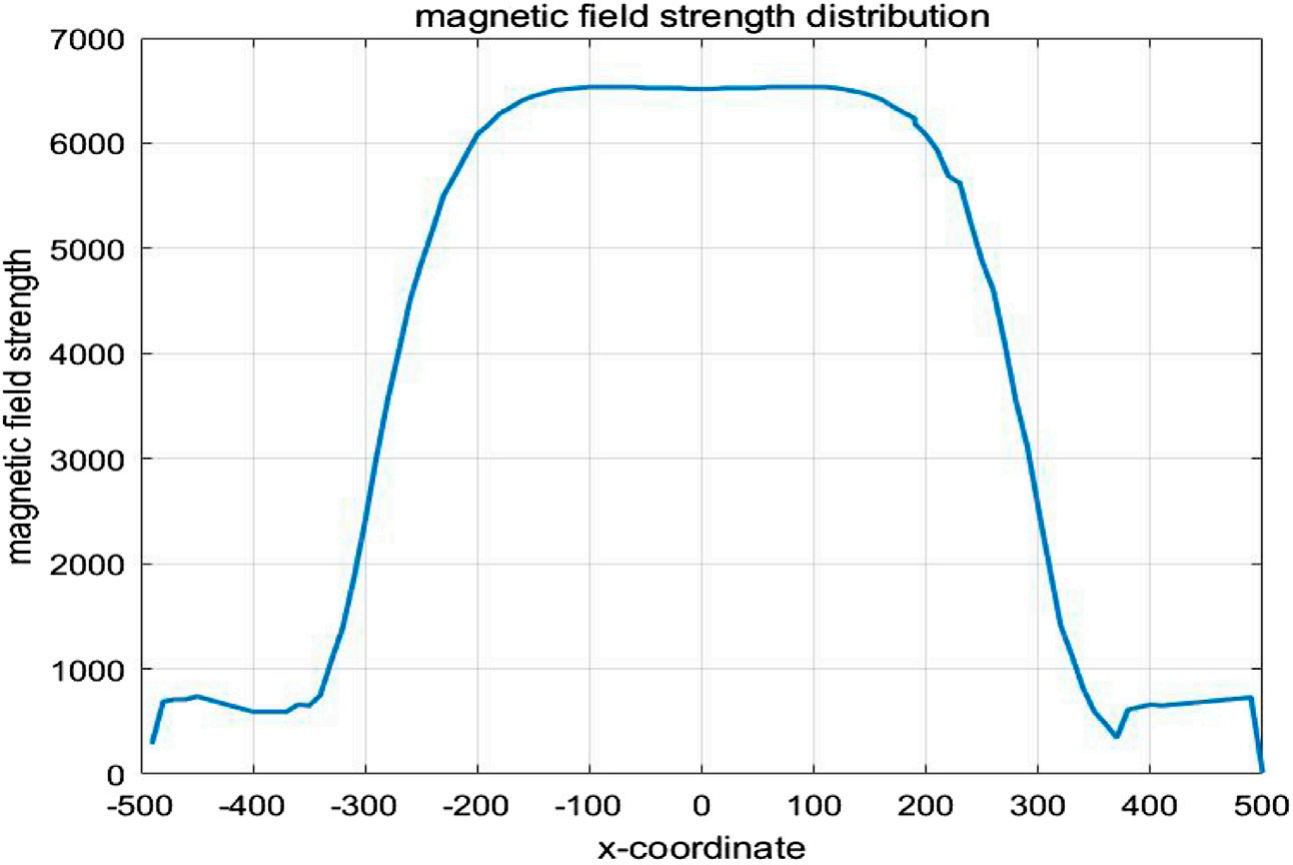
Magnetic field induction strength distribution along the x-axis.

The one-dimensional magnetic field strength distribution at different positions along the x-axis in the middle of the model is shown in Figure 9. At x = −140 mm,the magnetic field strength is equal to that at x = 140 mm, approximately 6682 A/m. At x = 0 mm,the magnetic field strength is measured as 6670 A/m. It can be seen that within the range of x = −140 mm to x = 140 mm, the amplitude change in magnetic field strength is about 0.18%, tending to stabilize.

### 4.2 Spatial electric field distribution

**FIGURE 10:**
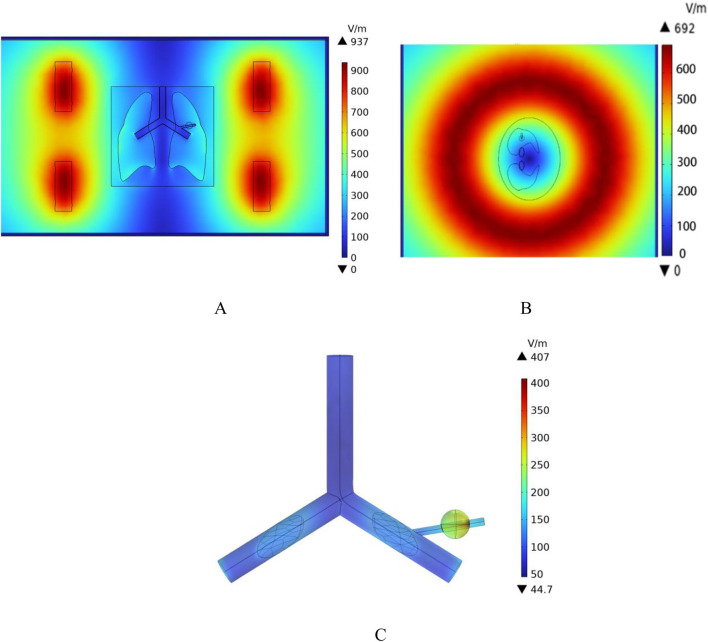
Spatial electric field distribution. **(A)** x-y Plane electric field distribution **(B)** z-x Plane electric field distribution **(C)** Blood vessel wall and tumor region electric field distribution.

According to the results in Figure 10, the distribution of induced electric field strength at different angles of the model can be observed. In this model, the dielectric constant of air is set to 1. The sample referenced in this study for simulation modeling is the human lung in its inflated state. Since the lung is filled with air in this state, its dielectric constant and electrical conductivity are significantly lower than those of the torso and other tissues. As a result, the induced electric field strength on the outer surface of the lung is notably higher than that within the lung tissue. Among them, the induced electric field strength in the tumor region is 240 V/m, while the induced electric field strength on the blood vessel wall intersecting with the tumor is 200 V/m.

### 4.3 Flow field distribution

**FIGURE 11:**
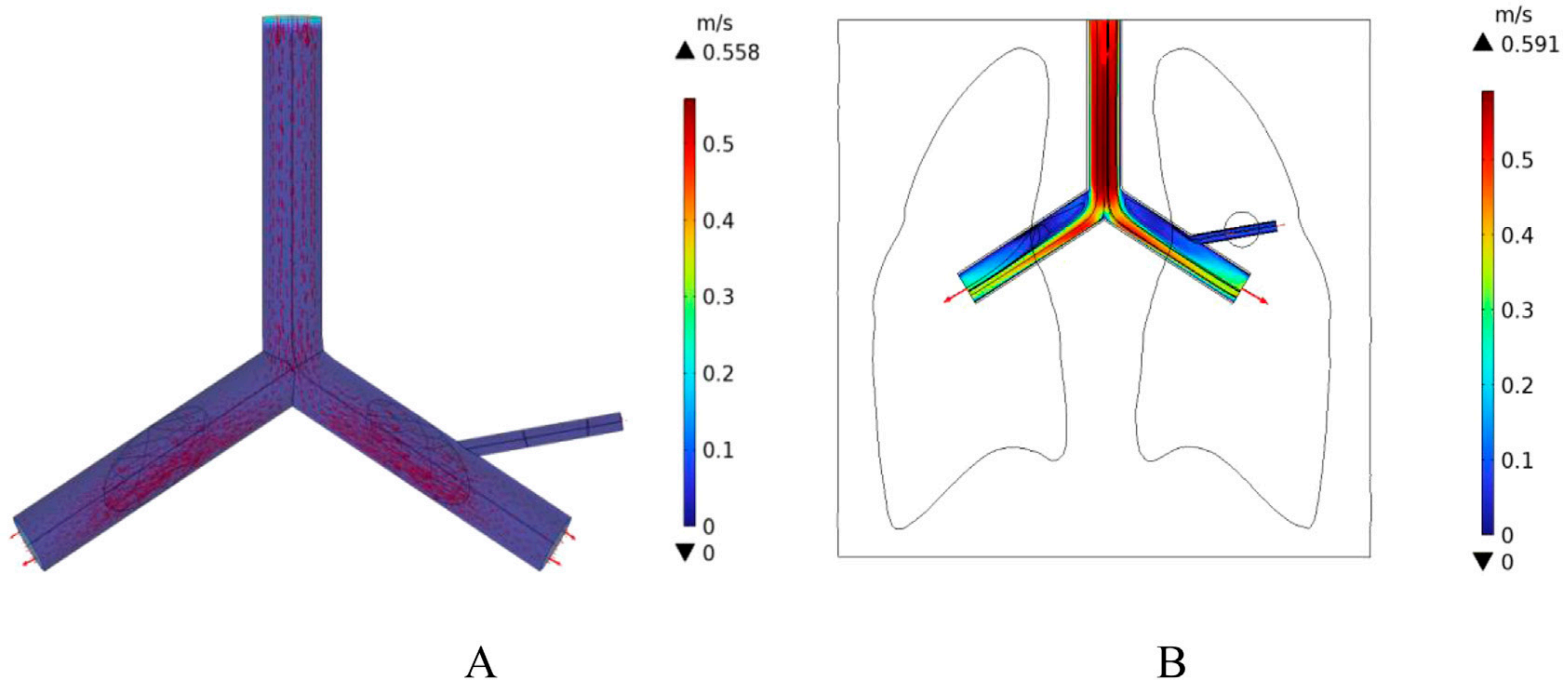
Distribution of blood flow velocity under normal flow speed conditions **(A)** Three-dimensional blood flow velocity diagram **(B)** Blood flow velocity distribution in the x-y plane.

In this study, a cylindrical rigid pipe was used to simulate the blood vessels, and blood flow within the vessels was treated as laminar flow, with blood considered as an incompressible Newtonian fluid. According to the data from reference (Ku, 1997), the dynamic viscosity of arterial blood is μ = 4.2 × 10^−3^ Pa s, and its density is ρ = 1,050 kg/m^3^. In COMSOL Multiphysics, a laminar flow physics interface was introduced, with an initial velocity of 0.5 m/s set for the large blood vessels, which is the typical reference velocity for blood flow in the human pulmonary artery (Iasiello et al., 2017). At the same time, the left pulmonary artery, right pulmonary artery, and the distal end of the right pulmonary branch artery were set as outlets, with static environmental pressure applied at these exit conditions to simulate the real blood flow state in human lung blood vessels. The velocity field distribution in the pulmonary blood vessels was calculated, as shown in Figure 11. According to the results in Figure 11, the blood flow velocity at the distal end of the right pulmonary branch artery is about 0.3 m/s, while the blood flow velocity in the right pulmonary branch artery associated with the tumor is 0.12 m/s.

**FIGURE 12:**
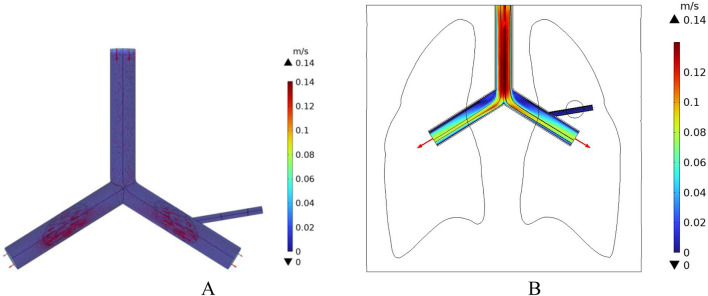
Blood flow velocity distribution at low flow velocity **(A)** 3D Blood flow velocity diagram **(B)** x-y Plane blood flow velocity distribution.

In addition, the initial velocity of the pulmonary artery is set at a low flow speed to compare the intensity of the heat sink effect caused by different blood flow velocities. The distribution of blood flow velocities in the vessels is shown in Figure 12. The maximum flow velocity in the pulmonary artery is 0.14 m/s, and the blood flow velocity in the right pulmonary artery near the tumor region is 0.02 m/s.

### 4.4 Temperature distribution

Using the COMSOL Multiphysics finite element software, the Bioheat Transfer Module and the Laminar Flow Module were employed to solve the temperature distribution of the model by coupling the non-isothermal flow interface with both physical fields. In this study, the magnetic fluid was injected into the pulmonary tumor tissue using the direct injection method, assuming the magnetic fluid is uniformly distributed after injection into the tumor. The initial temperature of all biological tissues was set to 37 °C, and the heating time was set to 500 s.

The calculated magnetic field strength of 6675 A/m in the therapeutic region (as obtained in Section 4.1) was substituted into Equation 13, resulting in a magnetic fluid heating power P0 = 664,360 W/m3. This heating power was then used as an external generalized heat source in the Bioheat Transfer Module for further calculations.

#### 4.4.1 Temperature distribution without blood vessel distribution

**FIGURE 13:**
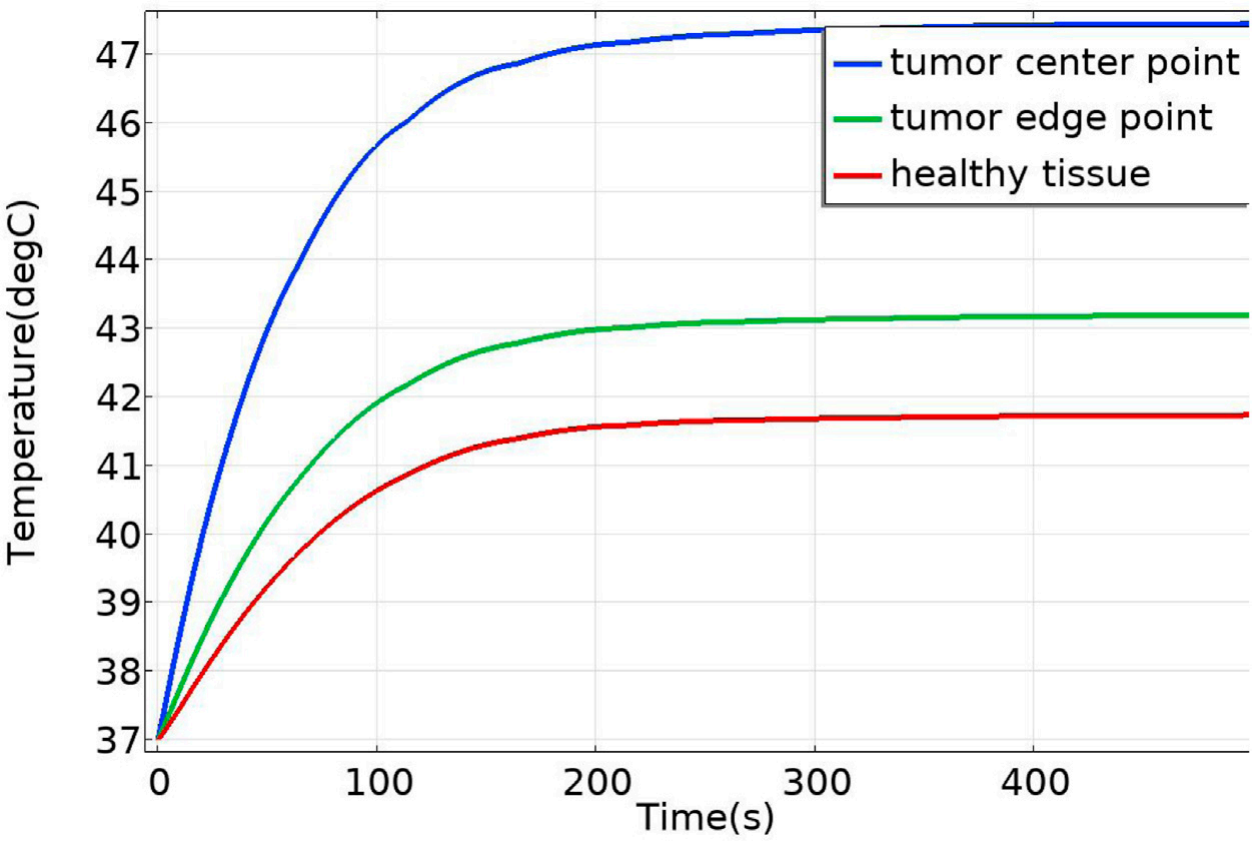
Temperature change over time without vascular distribution.

**FIGURE 14:**
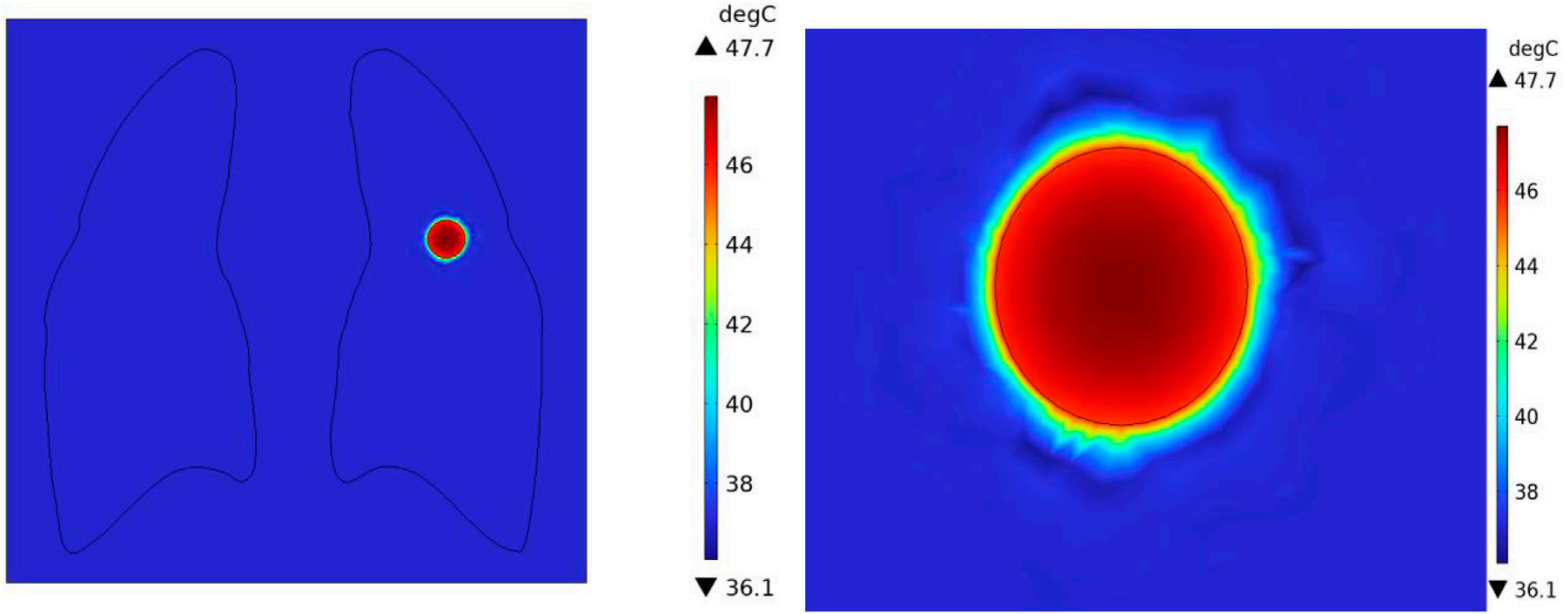
Temperature distribution in the x-y Plane without vascular distribution.

**FIGURE 15:**
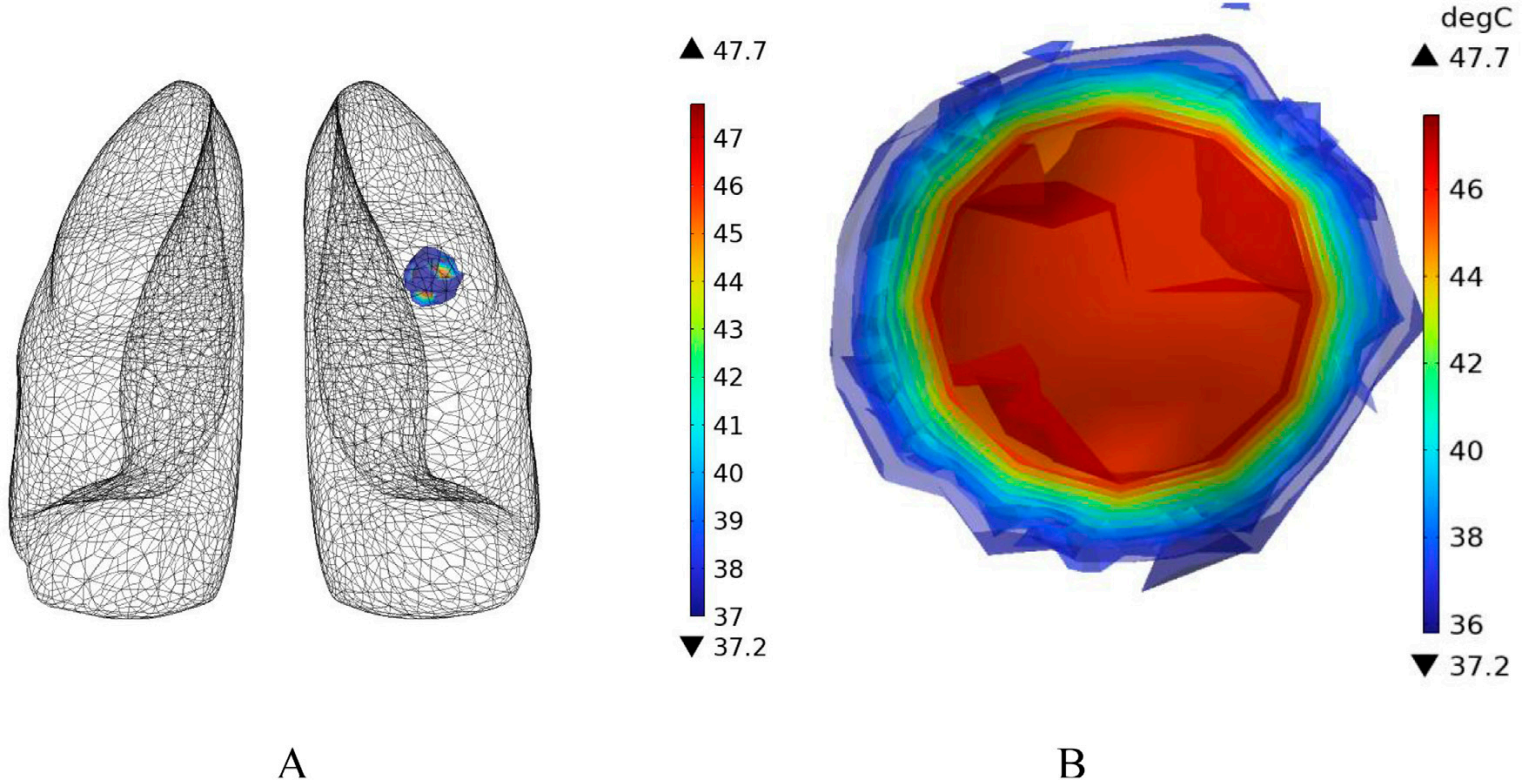
Three-dimensional temperature distribution of the model with no vascular distribution **(A)** overall temperature distribution of the model **(B)** temperature distribution profile of the tumor region.

In the case of no vascular distribution, the temperature field variations over time are shown in Figure 13, which demonstrates that the temperatures in different regions of the model stabilize and reach dynamic equilibrium after 300 s. Figure 14 shows the temperature field distribution at t = 400 s in the x-y plane at the tumor location. Figure 15 shows the temperature distribution map of the overall model and the tumor region cross-section. It can be observed that the highest temperature at the tumor center reaches 47.7 °C.

Additionally, temperature-time curves for the tumor edge and the boundary between tumor and healthy tissue under the heating of the Helmholtz coil were plotted. It can be seen that after 300 s, both points gradually reach a stable state. The highest temperature at the tumor edge is approximately 42.17 °C, while the temperature at the tumor-healthy tissue interface is about 41.72 °C. In magnetic induction hyperthermia, it is generally required that the tumor tissue reaches 42 °C or higher to induce tumor cell death, while the temperature of healthy tissue must not exceed 42 °C to avoid damaging healthy tissue cells. Based on the solved temperature field distribution, it can be concluded that the current setup meets the temperature requirements for magnetic induction hyperthermia.

#### 4.4.2 Effect of blood flow velocity on temperature distributionn

In an ideal scenario, the target area for magnetic induction hyperthermia treatment would be a uniform, homogeneous tissue. However, in reality, this is often not the case. Oncology research indicates that tumors typically appear around blood vessels and may develop their own complex vascular networks. The complex vascular distribution around tumors and the uncertainty of blood flow make clinical treatment more complicated. This is because the presence of blood vessels affects drug delivery, the effectiveness of hyperthermia, and the tumor’s microenvironment. Therefore, we must consider cases where blood vessels are present in tumors, and even cases where blood vessels directly cross through the tumor tissue region.

By coupling the flow field distribution solved in Section 4.3 for blood flow in the pulmonary vessels, we continue to input the magnetic fluid heating power P0 = 664,360 W/m3 into the lung tumor model with blood vessel distribution. The temperature field distribution in the model is then solved in COMSOL.

**FIGURE 16:**
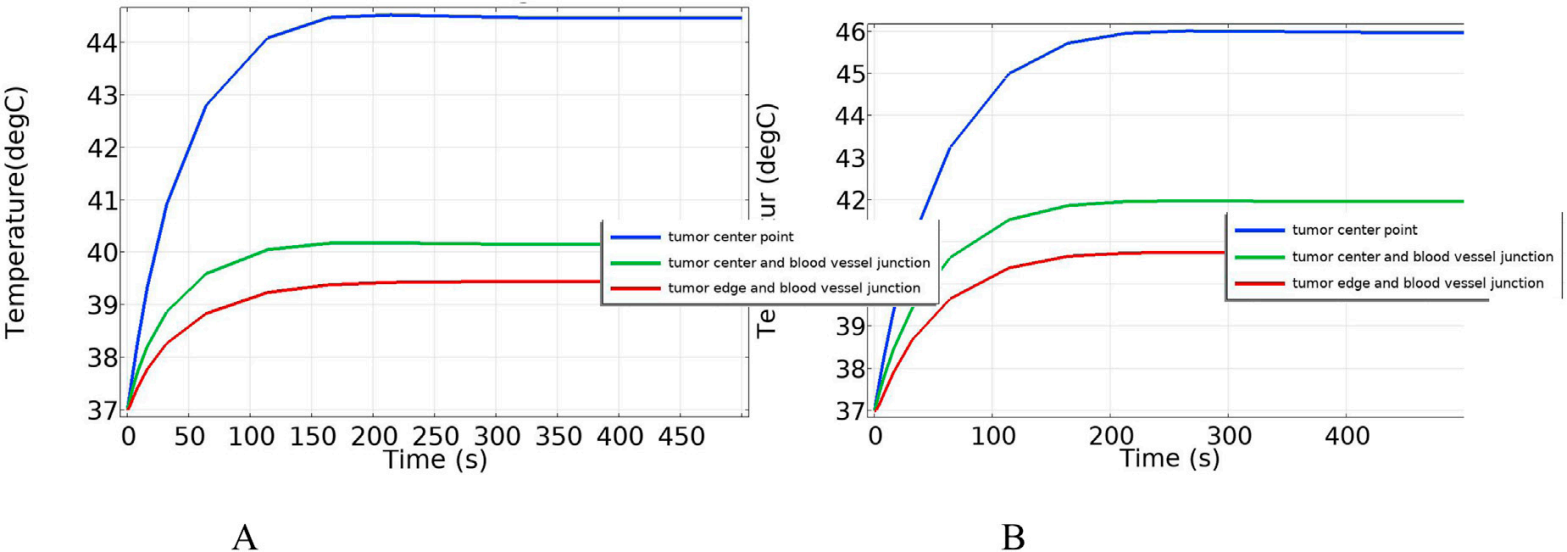
Temperature change over time with blood vessel distribution **(A)** Normal blood flow velocity **(B)** Low blood flow velocity.

**FIGURE 17:**
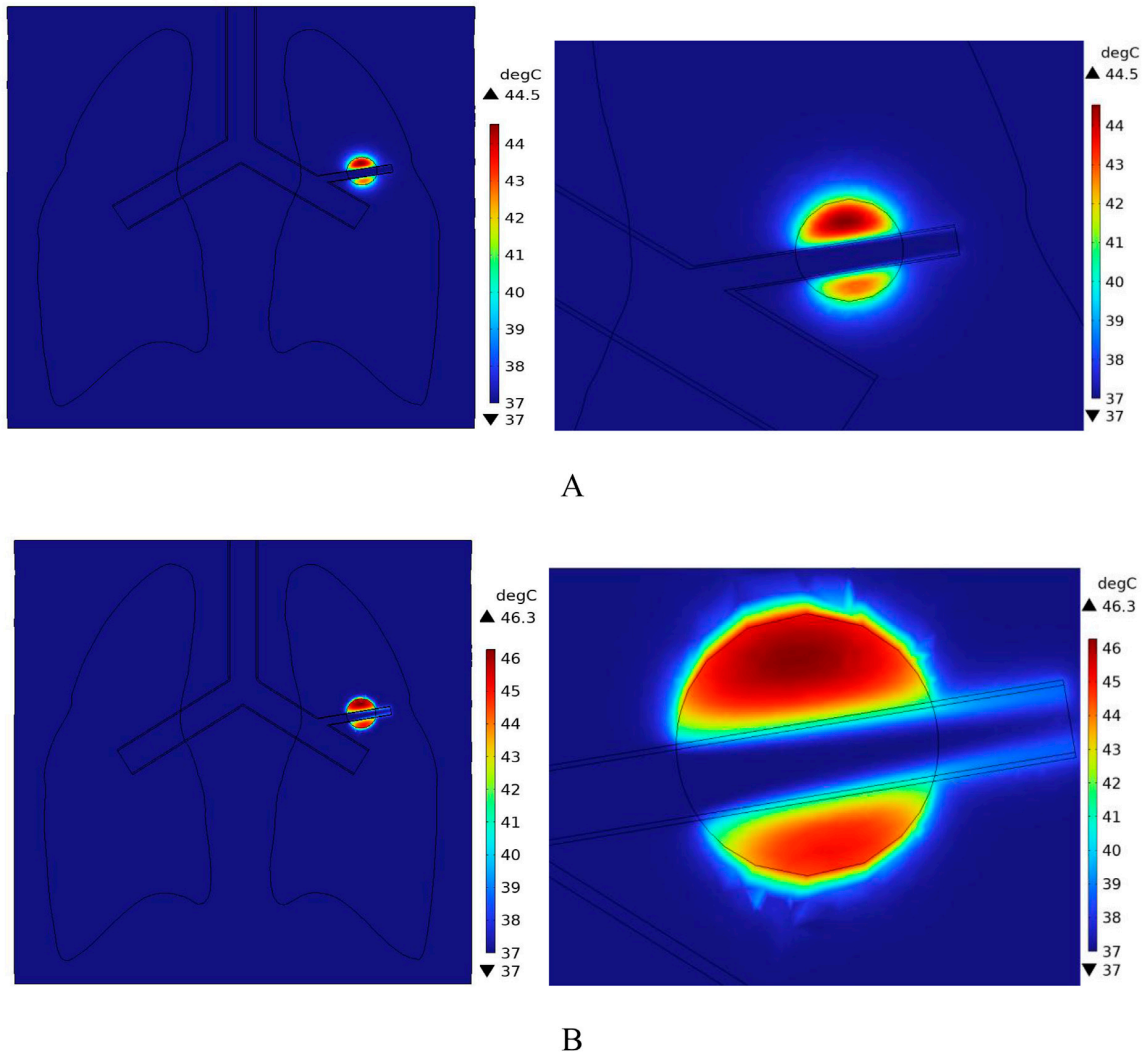
Temperature field distribution in the x-y plane with blood vessel distribution **(A)** Temperature distribution at normal flow velocity **(B)** Temperature distribution at low flow velocity.

Figure 16 shows the temperature-time curves for three points: the tumor center, the tumor-vessel interface, and the tumor edge-vessel interface at different blood flow velocities. From the results, it can be seen that after 250 s of heating, the temperatures of all three points gradually stabilize. The temperature field distribution at t = 300th = 300th = 300 s for the tumor in the x-y plane is shown in Figure 17. According to the results in Figure 17A, when blood vessel distribution is present and blood flow is at the normal velocity, neither the tumor-vessel interface nor the outer edge points reach 42 °C, which is the required temperature for magnetic induction hyperthermia. The tumor center reaches a maximum temperature of about 44.5 °C, while the tumor center at the blood vessel interface stabilizes at about 41.17 °C. Compared to the corresponding points in the model with no vascular distribution in Section 4.4.1, the maximum temperature in the tumor region decreases by about 6.7%. The temperature at the tumor edge-vessel interface stabilizes at around 39.45 °C, a decrease of approximately 6.5%.

**FIGURE 18:**
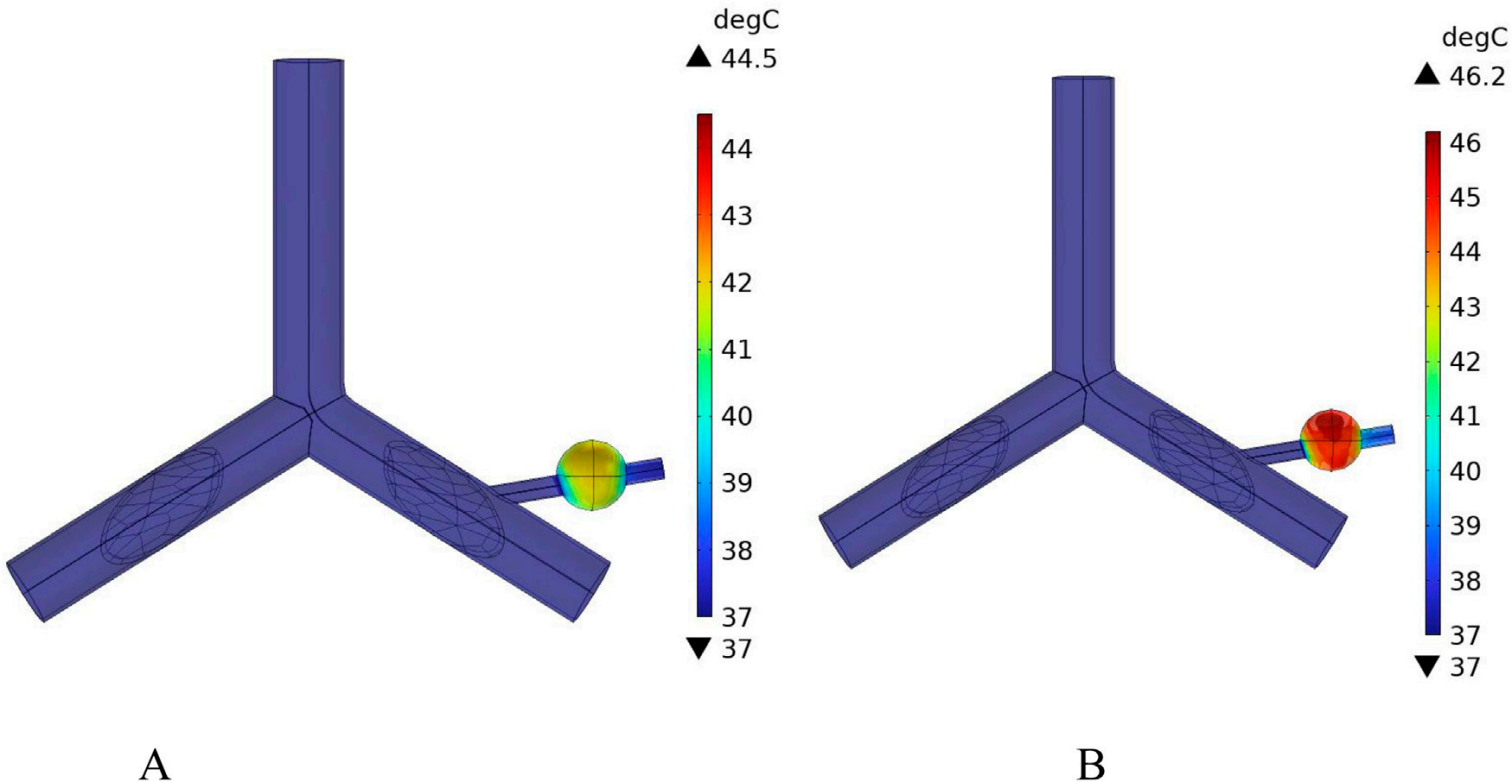
Temperature distribution in the blood vessel and tumor region **(A)** Model temperature distribution at normal flow velocity **(B)** Model temperature distribution at low flow velocity.

**FIGURE 19:**
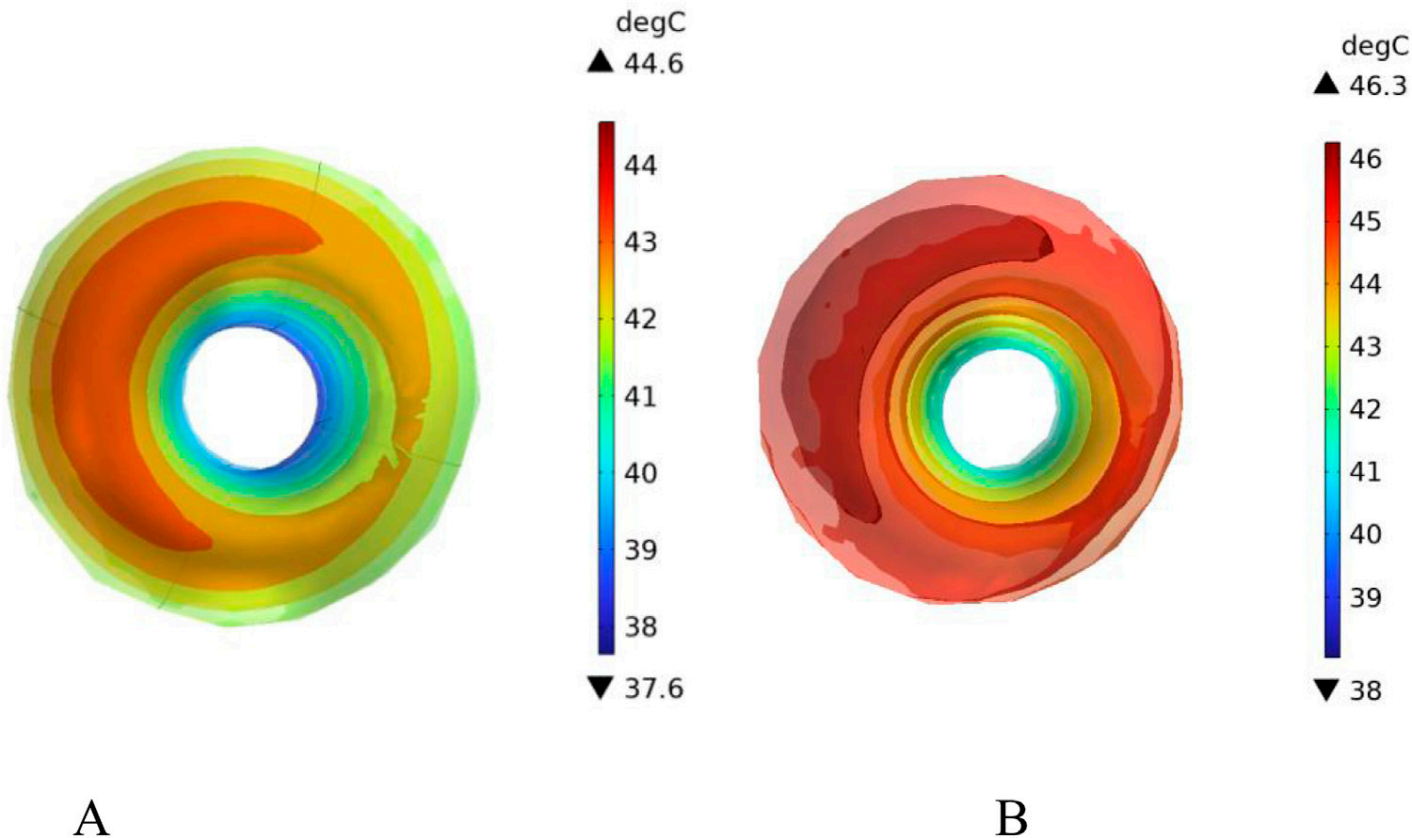
Tumor region profile temperature distribution **(A)** Tumor region temperature distribution at normal flow velocity **(B)** Tumor region temperature distribution at low flow velocity.

Figure 18 shows the temperature distribution in the blood vessel and tumor region of the model under different flow velocity states with blood vessel distribution. Referring to the flow field distribution of the model at low flow velocity in Section 4.3, the blood flow velocity in the right pulmonary branch artery passing through the tumor tissue region is 0.02 m/s. According to Figure 17B, compared to the temperature distribution of the model under normal flow velocity, the maximum temperature in the tumor region reaches 46.3 °C. Combining the temperature distribution diagram in the tumor region profile shown in Figure 19, under the low flow velocity condition, all areas except for the tumor edge-vessel interface can reach 42 °C or higher.

#### 4.4.3 Effect of vessel diameter on temperature distribution

Studies on the vascular system of pulmonary tumors have revealed that the blood vessel diameters exhibit a cross-scale distribution (Gong et al., 2024). Therefore, it is necessary to investigate the influence of different vessel diameters on the temperature distribution during magnetic induction hyperthermia.

**FIGURE 20:**
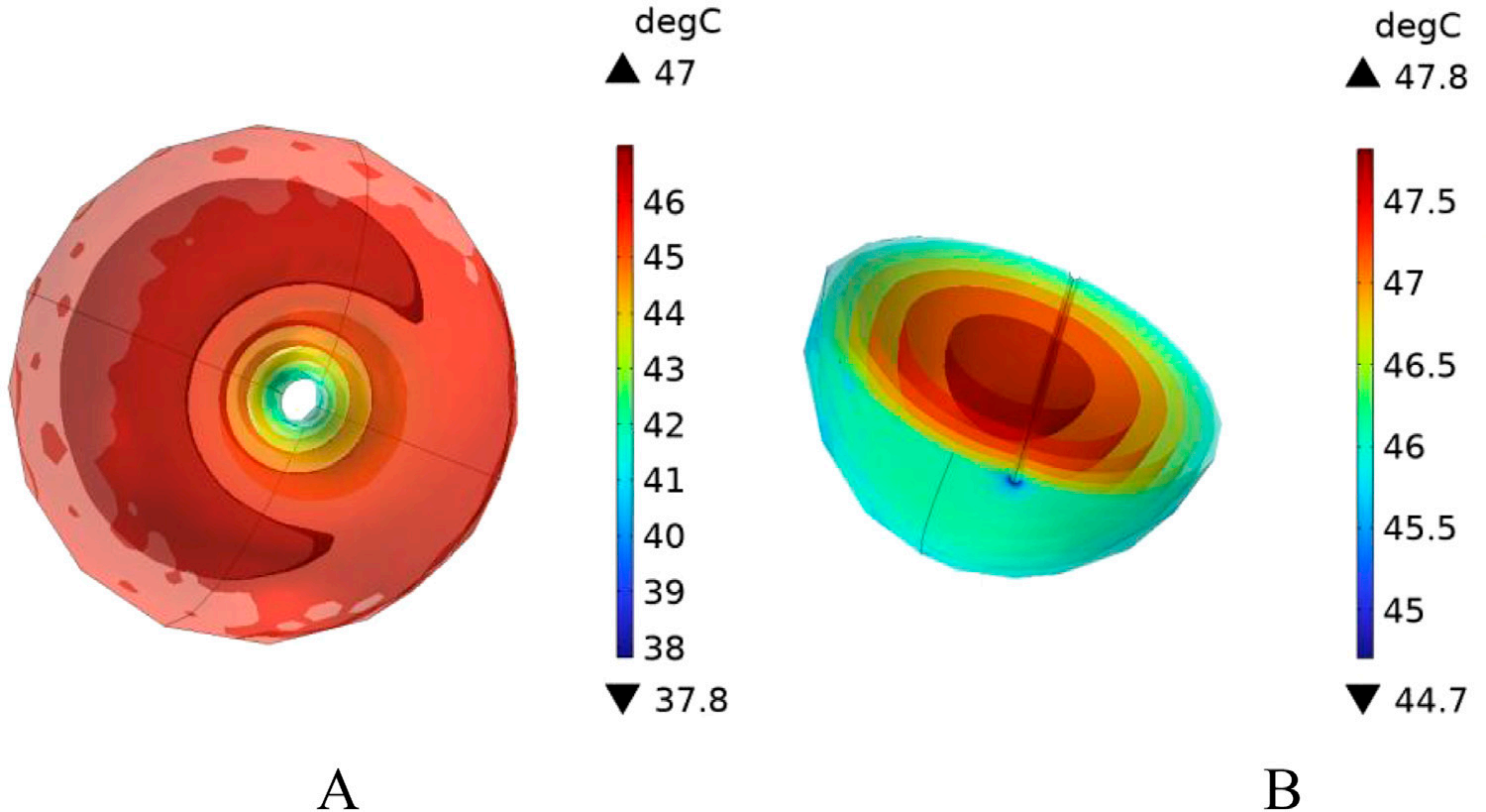
Presents the cross-sectional temperature distribution of the tumor region under different blood vessel diameters: **(A)** vessel diameter of 2 mm, and **(B)** vessel diameter of 0.5 mm.

In this section, additional simulations are conducted with vessel diameters set to 2 mm and 0.5 mm, respectively. Figure 20 illustrates the cross-sectional temperature distribution in the tumor region under these conditions. As shown in the figure, when the vessel diameters are 2 mm and 0.5 mm, the temperatures at all locations within the tumor region reach or exceed 42 °C, satisfying the thermal requirements for effective magnetic induction hyperthermia.

**FIGURE 21:**
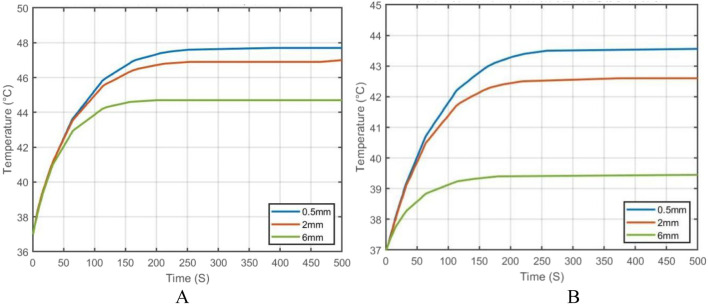
Temperature evolution at characteristic points in the tumor under different blood vessel diameters: **(A)** Temperature variation at the tumor center (maximum temperature point); **(B)** Temperature variation at the tumor edge near the vessel interface (minimum temperature point).

To further analyze the effect of vessel diameter, the temperature evolution over time was recorded at the tumor center (maximum temperature point) and at the tumor boundary near the vessel interface (minimum temperature point). The results are plotted in Figure 21. As shown, when the vessel diameter is 2 mm, the maximum temperature in the tumor region reaches approximately 47 °C, while the minimum temperature stabilizes around 42.5 °C. Compared to the case without vascular structures, the maximum and minimum temperatures decrease by only 1.5% and 1.6%, respectively, under the same heating power.

When the vessel diameter is further reduced to 0.5 mm, the maximum and minimum temperatures at the tumor center and edge reach 47.7 °C and 43.2 °C, respectively. These results indicate that when the vessel diameter is smaller than 0.5 mm, the heat sink effect induced by blood flow has negligible impact on the efficacy of magnetic induction hyperthermia at the given power level.

## 5 Discussion

Magnetic Induction Hyperthermia (MIH) has received increasing attention in the field of tumor physical therapy in recent years due to its good penetration depth, biocompatibility, and highly controllable thermal effects. With the continuous optimization of magnetic nanoparticle materials and the development of heating device structures, this technology has demonstrated outstanding advantages in targeted precise treatment and local temperature control. This study investigates the impact of blood flow on the temperature field distribution during MIH treatment. By introducing a human thoracic and lung tissue model, along with vascular and blood flow models, and using the coupling method of electromagnetic and heat transfer models, a relatively complete simulation system for human lung tissue was constructed. The COMSOL Multiphysics 6.2 simulation platform was used to systematically analyze the spatial electromagnetic field distribution, flow field changes, and thermal field evolution.

The study shows that using a Helmholtz coil with 280 turns, excited by 10A and 100 kHz current, a uniformly distributed magnetic field with consistent direction and strength is generated in the axial region. Without introducing a metallic magnetic core, a uniform magnetic field of 8.38 mT can still be achieved in the center, meeting the external excitation conditions required for effective magnetic nanoparticle activation.

In this study, the magnetic fluid was directly injected into the tumor region, with the volume fraction of the magnetic fluid set to 0.003. It was assumed that the magnetic nanoparticles were uniformly distributed within the tumor. The simulation results indicate that, in the absence of blood flow, the temperature at the tumor center can quickly rise to 47.7 °C within 300 s, and the temperature in the peripheral region stabilizes above 42 °C. All tumor areas meet the temperature threshold for thermal apoptosis of cells, while the temperature of healthy tissue remains below 42 °C, indicating good heating performance.

However, when the right pulmonary branch artery was added to the model, and blood flow was simulated within the vessel using an incompressible Newtonian fluid to mimic real blood flow, significant changes in the thermal field distribution were observed. The introduction of blood vessels rapidly dissipates heat along the direction of blood flow, forming a clear temperature gradient at the tumor-vessel interface. The center temperature decreases to 44.5 °C, and the peripheral region temperature drops to 39.4 °C. Compared to the model without blood flow, the maximum temperature decreased by more than 3.2 °C, and the temperature in the peripheral region decreased by about 6.5%. This suggests that blood flow has a strong heat-sink effect during tumor hyperthermia, significantly reducing the temperature rise efficiency in the treatment area and affecting the actual effectiveness of MIH.

To further investigate the influence of blood flow velocity and vessel diameter on the intensity of the heat sink effect, a low-velocity blood flow model and vessels with varying diameters were introduced as control conditions. The results indicate that blood flow velocity significantly affects the heat sink effect: when the intravascular velocity decreases from 0.13 m/s to 0.02 m/s, the maximum temperature in the tumor region rises to 46.2 °C, and the minimum temperature at the tumor periphery increases to 40.9 °C, with approximately 90% of the tumor volume reaching the therapeutic threshold. Additionally, smaller vessel diameters result in weaker heat sink effects. When the vessel diameter is reduced to 0.5 mm, the temperature in both the tumor center and peripheral regions nearly returns to the level observed in the absence of blood vessels, indicating a significantly diminished heat sink effect. These findings suggest that vascular anatomical structures should be carefully considered in clinical applications to optimize hyperthermia treatment strategies.

This study systematically investigated the influence of blood flow on temperature distribution during magnetic induction hyperthermia (MIH) of pulmonary tumors. The results confirmed that vascular structures and flow dynamics exert a substantial heat sink effect, which markedly reduces the therapeutic temperature in the tumor core and peripheral regions. These findings are consistent with prior reports that blood perfusion can significantly alter local hyperthermia efficacy, while also extending the current understanding by quantifying the effects of both blood flow velocity and vessel diameter on intratumoral thermal profiles.

Compared with the hypotheses outlined in the introduction, our simulations confirmed that increased flow velocity and larger vessel diameters exacerbate heat dissipation, thereby compromising the uniformity and efficacy of MIH. Conversely, reduced flow velocity or smaller vessel diameters mitigate this effect, enabling more tumor regions to reach the therapeutic threshold of 42 °C. These results support the hypothesis that blood flow plays a pivotal role in determining MIH treatment outcomes, but also highlight potential scenarios where its negative impact may be minimized.

Clinically, these results suggest that patient-specific vascular anatomy and perfusion rates must be carefully considered when designing MIH treatment protocols. For example, tumors adjacent to large vessels or under high-perfusion conditions may require enhanced excitation currents (Tang et al., 2023), optimized coil geometries, or improved nanoparticle delivery strategies to achieve adequate heating (Latorre and Rinaldi, 2009). These insights underscore the importance of integrating vascular parameters into individualized treatment planning for pulmonary tumors.

It is also important to acknowledge certain limitations of this study. The blood was modeled as a Newtonian fluid, and magnetic nanoparticles were assumed to be uniformly distributed, which may not fully capture the complexity of *in vivo* conditions. Additionally, solid–fluid thermal interactions were simplified by treating vascular walls as rigid, neglecting possible deformations. Addressing these limitations in future research will enhance the robustness and translational potential of simulation outcomes.

Future work should validate the present computational findings through *in vitro* or *in vivo* experiments, as well as explore heterogeneous nanoparticle distributions and non-Newtonian blood flow models to better approximate physiological conditions. Furthermore, technical refinements in coil design and adaptive field control may offer promising pathways to overcome heat sink effects and improve clinical feasibility.

This study provides a theoretical foundation for the quantitative analysis of the impact of blood flow in magnetic induction hyperthermia and offers important references for the clinical development of personalized heating strategies and the optimization of magnetic field parameters. With the advancement of simulation technologies and intelligent magnetic field control, magnetic induction hyperthermia is expected to play a greater role in solid tumor treatment, achieving safer, more effective, and reproducible tumor control goals.

## Data Availability

The original contributions presented in the study are included in the article/supplementary material, further inquiries can be directed to the corresponding author.
